# Phenotyping clonal populations of glioma stem cell reveals a high degree of plasticity in response to changes of microenvironment

**DOI:** 10.1038/s41374-021-00695-2

**Published:** 2021-11-15

**Authors:** James A. Innes, Andrew S. Lowe, Raquel Fonseca, Natasha Aley, Tedani El-Hassan, Myrianni Constantinou, Joanne Lau, Ayad Eddaoudi, Silvia Marino, Sebastian Brandner

**Affiliations:** 1 Department of Neurodegenerative Disease, Queen Square Institute of Neurology, University College London, Queen Square, London WC1N 3BG UK; 2 Blizard Institute, Barts and The London School of Medicine and Dentistry, Queen Mary University London, London, E1 2AT UK; 3 Zayed Centre for Research Into Rare Disease in Children, UCL Great Ormond Street Institute of Child Health, University College London, London, WC1N 1DZ UK

**Keywords:** Mechanisms of disease, Viral tracing, Cancer stem cells

## Abstract

The phenotype of glioma-initiating cells (GIC) is modulated by cell-intrinsic and cell-extrinsic factors. Phenotypic heterogeneity and plasticity of GIC is an important limitation to therapeutic approaches targeting cancer stem cells. Plasticity also presents a challenge to the identification, isolation, and propagation of purified cancer stem cells. Here we use a barcode labelling approach of GIC to generate clonal populations over a number of passages, in combination with phenotyping using the established stem cell markers CD133, CD15, CD44, and A2B5. Using two cell lines derived from isocitrate dehydrogenase (IDH)-wildtype glioblastoma, we identify a remarkable heterogeneity of the phenotypes between the cell lines. During passaging, clonal expansion manifests as the emergence of a limited number of barcoded clones and a decrease in the overall number of clones. Dual-labelled GIC are capable of forming traceable clonal populations which emerge after as few as two passages from mixed cultures and through analyses of similarity of relative proportions of 16 surface markers we were able to pinpoint the fate of such populations. By generating tumour organoids we observed a remarkable persistence of dominant clones but also a significant plasticity of stemness marker expression. Our study presents an experimental approach to simultaneously barcode and phenotype glioma-initiating cells to assess their functional properties, for example to screen newly established GIC for tumour-specific therapeutic vulnerabilities.

## Abstract (web summary)

The authors barcoded glioma-initiating cells (GIC) using combinations of virally encoded fluorophores. GIC show a strong tendency to form clonal populations over as few as two passages. Combined with stem cell marker phenotyping and computational analysis, they could trace the fate of such populations. This model presents an approach for rapid assessment of newly established GIC to assess tumour-specific vulnerabilities.

## Introduction

Glioblastoma-initiating cells (GIC) can recreate aspects of inter and intra-tumour cancer stem cell (CSC) heterogeneity. Cell identity can be modulated by cell-intrinsic factors such as genetic, epigenetic, and metabolic factors, and further diversified by cell-extrinsic factors, created by the microenvironment, niche factors, and the host immune system^1^. Genetic heterogeneity within tumour bulk can evolve through selective pressure such as therapy, favouring emergence of clones with resistant properties^2^. Advances in the understanding of phenotypic heterogeneity have challenged the definition of CSC differentiation as a unidirectional and irreversible hierarchy^3^. Such plasticity is key to the understanding of pathogenesis of tumours, where phenotypic shift can occur during initiation, progression, and selection of clones during development of therapy resistance^4^.

A proportion of cells in a tumour bulk is thought to have CSC properties, i.e., the potential to drive tumour growth but they may not do so in the absence of a permissive environment, or they may be killed by extrinsic factors such as immune cells or by a therapy. Slowly proliferating clones may be at competitive disadvantage to highly proliferative clones, but when transferred into a permissive environment lacking restrictive factors such as for example stromal cells^5^, they may expand and form a predominant population, permitting the same cells to form tumours after transplantation. The identification and isolation of CSC populations to a high degree of purity using combinations of CSC markers remains a significant challenge due to their plasticity, and there are no established combinations of markers that can demonstrate a high degree of “CSC purity”^6^. These difficulties however could explain the variability between patient samples or the intrinsic adaptability of stem cells as a response to variations in experimental conditions.

A number of markers have been associated with stemness properties of CSC, and amongst them CD133, CD15, CD44, and A2B5 have been most widely used. CD133 is a cell surface protein that served as neural stem cell marker^7^ and was initially thought to delineate GIC from tumour bulk populations^8,9^. As few as 300 xenotransplanted CD133+ cells, but not 100,000 CD133− cells can form tumours, and have prompted the hypothesis of a hierarchical system of self-renewing GIC^10^. However, subsequent studies found CD133+ and CD133− cellular fractions shared many of the same stem cell characteristics^11–13^ and indeed a potential for CD133- cells to form tumours containing CD133+ cells, which may be explained by fluctuation of CD133 expression during cell cycle^14^. Another marker thought to characterise cells with tumour initiating potential is CD15/SSEA1/LeX^15^, which is expressed in cells of the proneural glioma subtype. Initially discovered as an extracellular matrix-associated carbohydrate expressed by embryonic stem cells, it was later characterised as a surface marker expressed on medulloblastoma^16^ and GIC^17^. However, later studies have suggested that CD15 is non-selective for a distinct population in glioblastoma^18^. CD44 has also been proposed as possible marker, potentially associated with specific subgroups of GBM^17^, and reportedly CD44 expressing GIC have a strong propensity to generate xenografted tumours, compared to CD44-negative populations^19^. The A2B5 surface marker is named after an antibody clone recognising a ganglioside on the surface of glial progenitor cells^20^, and this A2B5-immunoreactive ganglioside is differentially expressed on GIC^12,21^.

Multiple studies have demonstrated the utility of combining these surface markers to characterise functionally distinct populations of glioblastoma stem cells^9,10,12,19,22^, but at the same time it has been recognised as a caveat that these markers can be modulated by changing the microenvironment^1,23^. A recent study^24^ used the markers CD133, CD44, CD15 and A2B5 to characterise GBM intratumoural heterogeneity and to show a striking plasticity of the expression of these stemness markers. Sixteen purified “phenotypes” of CSC could be isolated and after expansion, each of those could give rise to populations corresponding to the other 15 phenotypes. These phenotypes (which include cells negative for all markers) did not show distinct properties in cell assays such as self-renewal and doubling time which underpins the notion that marker-defined phenotypes do not describe cells at the apex of a hierarchy but instead a plasticity contributed to by the microenvironment.

Multicolour labelling using lentiviral gene ontology (“LeGO”) vectors has proven a useful tool to “barcode” cell populations and to identify emerging clonal populations. First described as RGB (red, green, blue) marking using overlapping expression of three lentiviral vectors^25–27^, this approach has been subsequently refined to accommodate vectors encoding six distinct fluorescent colours, to optically barcode theoretically up to 41 different fluorophore combinations^28^. This methodology has been applied in vitro and in vivo to characterise clonal evolution in liver tumours^29^, osteosarcoma^30^, brain tumours in the context of inflammasome modulation^31^, mesenchymal stromal cells^32^, and in development^33,34^.

To address the relationship between surface-marker heterogeneity (phenotype) and clonality, an aspect that has not been widely studied in GBM, we have combined barcode labelling of GIC (enhanced blue fluorescent protein (EBFP) 2, T-Sapphire, Venus, and mOrange) with simultaneous capture of their surface-marker profiles for CD133, CD44, CD15, and A2B5. The importance of understanding heterogeneity in clonal dynamics, thought to be an outcome of fate decisions made by GIC, has been previously suggested^35^. We used this combined approach to study phenotypic plasticity of clonal populations during in vitro propagation in adherent culture and more complex three-dimensional organoid cultures to gain a better understanding if the plasticity of a cell population is attributed to an alteration of surface marker expression in a relatively stable genetically defined (barcoded) population of GIC, or a differential expansion of a heterogenous starting population. Such understanding is important to develop assays that can help systematically characterising GIC newly derived from patient material.

## Methods

### Human tissue resources and derivation of glioma-initiating cells

Fresh GBM tissue was sliced and triturated with a razor blade, dissociated with Accumax (Sigma, A7089) at 37 °C for 10 min and then filtered through a 70 µm cell strainer. Dissociated cells were plated on laminin-coated 6-well plates in NeuroCult NS-A Proliferation kit media (STEMCELL, 05751), heparin (2 lg/ml; Gibco 12587-010), mEGF (20 ng/ml, Preprotech, 315-09) and hFGF (10 ng/ml; Preprotech, AF-100-18B). Established cells were passaged when 70% confluent, detached using Accutase (Sigma, A6964). When necessary, cells were frozen in Stem Cell Banker (Amsbio ZENOAQ, 11890) and stored in liquid nitrogen as appropriate. Cell lines used in this study are primary lines derived from glioblastomas, which were molecularly characterised as IDH-wildtype glioblastomas according to diagnostic recommendations^36^. Primary tumours underwent Sanger sequencing for IDH1 and IDH2 gene mutations to confirm IDH-wildtype status and the presence of a TERT promoter mutation. Tumours also underwent methylation profiling^37,38^, and were confirmed to correspond to the methylation class glioblastoma, IDH-wildtype. Cell line G19 is derived from the glioblastoma of a 65 year old female. The tumour is wild-type on IDH1 and IDH2 genes and carries a TERT promoter mutation (C228T). The copy number profile shows MYCN and PDGFRA amplification, and chromosome 7 gain. Methylation profiling yielded the methylation class of glioblastoma, IDH-wildtype, subclass RTK I with a calibrated score of 0.97. Cell line G61 is derived from the glioblastoma of a 66-year-old female. The parent tumour showed no mutations on the IDH1 and IDH2 genes, and a TERT promoter mutation present (C228T). The copy number profile shows a CDKN2A/B homozygous deletion, chromosome 7 gain and chromosome 10 loss. Methylation profiling yields the methylation class glioblastoma, IDH-wildtype, subclass RTK II, with a calibrated score of 0.94. Demographic and molecular data of the parent tumours in Supplementary Table 1. Primary tumours and corresponding cell lines show nearly identical copy number profiles and matching methylation classes (ref. ^39^, Supplementary Fig. 1).

### Cell culture, sub-culturing, and organoid preparation

**Fig. 1:**
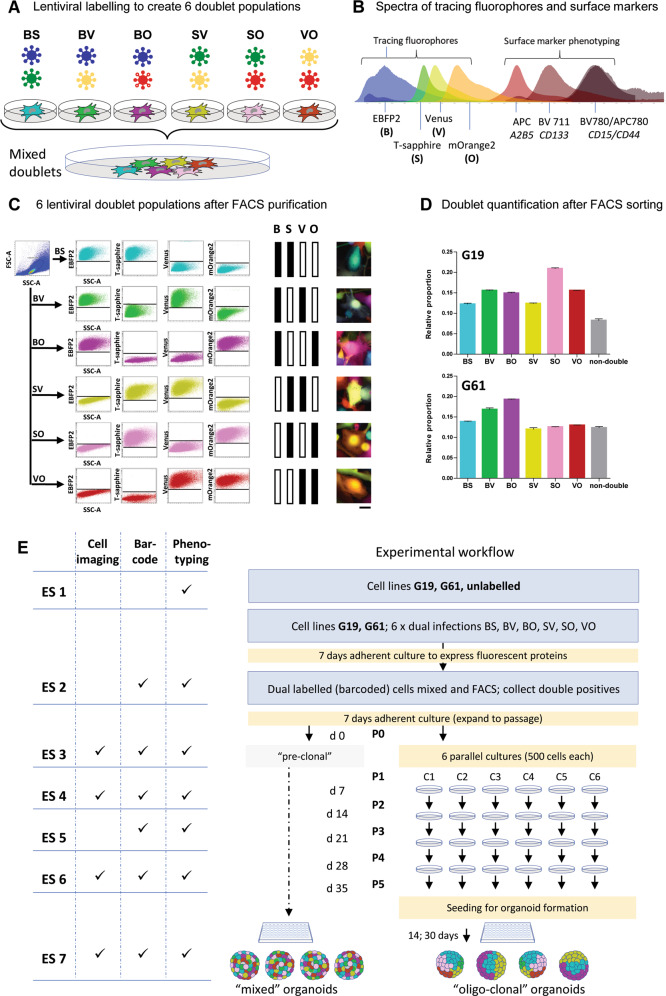
Lentiviral labelling protocol for barcoding of hGICs. **A** Combinations of four lentiviruses expressing EBFP2 (B [blue]), T-Sapphire (S), Venus (V) and mOrange2 (O) results in six populations of double labelled cells, BS, BV, BO, SV, SO, or VO. **B** Spectra of the fluorescent proteins and the surface markers, showing the fluorophores at the left of the spectrum, separated from the surface markers for expression phenotyping on the right of the spectrum. Spectral diagram from^49^. **C** Flow cytometry detection of 6 different labels in a mixed (*n* ~ 76,000) population derived from a cell culture one passage after FACS purification, eliminating single labelled cells. Axis dimensions set to demonstrate widespread of label detections in each colour group. The right panel shows examples of fluorescent images of double labelled cells. **D** Quantification of colour group frequencies from flow cytometry data (*n* = 3) for cell lines G19 and G61. **E** Experimental workflow encompassing all experimental steps including organoid formation. Experimental steps (“ES”) are indicated as ES 1–ES 6, to provide a generic reference in the text. Naïve cells (G19 and G61) were first analysed for surface marker expression (ES 1), and then transduced with lentivirus encoding two fluorescent labels and incubated to express them (ES 2), analysed by flow cytometry for clonality and surface marker expression (ES 3), propagated in six parallel cultures, over five passages (P1–P5) (ES 4, ES 5, ES 6), with three flow cytometry and surface marker measurements performed at ES 4, 5, and 6. Finally, two of the clonal cultures were processed to generate organoids (ES 7). In addition, mixed organoids were generated directly from mixed dual-labelled cells containing all label combinations before clonal segregation (left part of diagram). Scale bar in **A** corresponds to 10 µm.

Adherent cell cultures were maintained on laminin-coated plates in complete DMEM/F12 (Lonza, 12-719F) with B27 supplement, 1:1000 Heparin solution (Stemcell technologies, 07980) and 20 ng/ml human EGF and bFGF at 37 °C with 5% CO_2_ and atmospheric oxygen. To establish subcultures from 500 cells, after cellular detachment single-cell concentration was determined using an automated cell counter (Millipore, PHCC20060). Cells were then diluted into aliquots, to seed 500 cells each into individual wells of a 48-well plate. Subpopulations were expanded at P1 from 48-well into 12-well plates (Day 7) (Fig. 1E). From cultures that were seeded and monitored for growth, six cultures for G61 and G19 that grew out well were selected for further passaging and analysis. Glioblastoma organoids were established from 5000 primary hGICs, aliquoted from a suspension of 1 × 10^6^ cells/ml using an automated cell counter (Millipore, PHCC20060). hGIC suspension was prepared in a volume of 20 µl of a mixture of 80% Matrigel (Corning, 356234) and 20% single-cell suspension in DMEM/F12 complete medium as described^40^. To facilitate matrix gelling, the Matrigel-cell suspension was then incubated for 2 h at 37 °C in moulds created by pressing a piece of parafilm between two autoclaved U-bottom PCR plates. Then, organoids were removed from moulds and transferred to separate wells in a 12-well plate in appropriate cell culture medium with orbital shaking at 80 rpm. Organoids were harvested for imaging and flow analysis once they had reached maturity (30–40 days), judged by considerable light refraction and a darkened centre when viewed under a light microscope.

### Lentiviral vectors and barcoding of cells

Lentiviral gene ontology vectors (LeGOs) were used for stable transduction of target cells. The following fluorophores, cloned into the vector [LeGO-G2], were used: EBFP2 (blue; abbreviated as “B”; plasmid 14891; Addgene)^41^, T-Sapphire (violet excitable green; abbreviated as “S”)^42^, and mOrange2 (orange; abbreviated as “O”; plasmid 30175; Addgene) (Fig. 1B). LeGO vectors were packaged using the second-generation packaging vector psPAX2 (Addgene 12260) and the envelope vector pMD2.G (Addgene 12259) in HEK293T using lipofectamine 2000® (Thermofisher 11668027) according to manufacturer’s instructions. Virus was concentrated using Retro-X™ concentrator (Takara bio, 631455). Concentrated lentiviral titres (plaque-forming units) were calculated for each vector in line G61 as described previously^27^. For G19, the titre was determined for a single virus and the ratio between the G19 and G61 titres for this virus was calculated. This ratio was used to convert all experimentally determined G61 titres into corresponding values for G19. Viral barcoding was performed under the same conditions as titre calculations; 4 h viral incubation with 8 ng/µl polybrene in complete DMEM/F12. Six double transfections using the six combinations of the four fluorescent constructs (BS, BV, BO, SV, SO, VO) were performed (Fig. 1A). Viral titre data were used to calculate the volume of each viral preparation required to deliver equal amounts of viral particles and therefore achieve similar gene transfer efficiencies of the co-transfected vectors (Fig. 1D). Double labelled cells were further purified by FACS separation (FACS Aria III, BD Bioscience, Berkshire, UK) of double labelled cells from cells labelled with a single fluorophore (Fig. 1C, Supplementary Fig. 2).

### Single-cell sorting and clonality assay

G61 and G19 dual-labelled cells (BS, BV, BO, SV, SO, VO) were expanded in adherent culture, detached and dissociated to single-cell suspension (Accutase^®^, Sigma-Aldrich A6964) in flow cytometry pre-sort buffer (BD Biosciences, 5636503). Differentially labelled populations were gated (Supplementary Fig. 3) and single cells sorted into individual wells of a U-bottom 96-well plate using the FACS Aria III cell sorter. Cells were sorted into 50 μl GIC culture medium (see above) per well, and additional 50 μl fresh medium added at 1, 2, and 3 weeks of culture. Successful single-cell colony formation was determined after 4 weeks of culture. For G19, a neurosphere over the size of 40 μm were considered as successful colony and for G61, wells with over 100 adherent cells were considered as successful colony formation. Images were captured on a Zeiss LSM 710 confocal microscope through online fingerprinting using spectrally captured references for each fluorophore (EBFP2, T-sapphire, Venus and mOrange2) (Supplementary Fig. 3).

### Surface marker labelling and flow cytometry

Adherent cells were detached from plates using Accutase^®^ (Sigma-Aldrich A6964) and organoid sections were dissociated using Accumax^®^ (Invitrogen 00-4666-56) according to the manufacturers’ instructions. Detached single cells and dissociated organoids were washed 1× in flow cytometry staining buffer (Invitrogen, 00-4222-26) and organoids were passed through a 70 µm filter prior to Fc receptor blocking (14-9161-73) for 30 min at 4 °C in the dark. All blocking and staining steps were performed in flow cytometry staining buffer. Cells were incubated with conjugated antibodies for 30 min at 4 °C. Antibody targets for surface marker phenotyping were CD44 (Miltenyibiotec, 130-113-332), CD133 (BD,747641), A2B5 (Miltenyibiotec, 130-093-582) and CD15 (BD, 563838). Data acquisition was performed on a CytoFLEX-S (Beckman-Coulter, High-Wycombe, UK) cell analyser with data analysis done on CytExpert (Beckman-Coulter, High-Wycombe, UK) and Flowjo™. Filter sets and lasers used for data acquisition are listed in Supplementary Table 2. Gating strategy for phenotyping of doublet and clonal populations is shown in Supplementary Fig. 2. Gating strategy for quantification of marker profiles is shown in Supplementary Fig. 4. Enrichment of values for clonal analysis was done in two steps. (1) purification of doublet groups to ensure that only cells positive for two markers, and therefore having high degree of confidence these are clonal are dated. This on average removed ~20% (15–28%) and 15% (9–20%) of whole culture data points for G61 and G19 respectively. (2) purification of the doublet population to gating of individual clones (from the visible “clouds”, or “streaks”). Here, on average clones represent 73% (55–87%) and 65% (63–73%) of this purified doublet population for G61 and G19, respectively.

### Tissue sectioning and histology

Viable 30–40-day old organoids (tumour spheroids) suspended in culture medium were mixed 1:1 with 2% agarose at 45 °C. Wells of a 24-well plate served as a mould in which the organoid containing agarose/medium mixture was solidified. Moulds were trimmed and sectioned at 250 µm thickness on a vibratome^®^ 1500 sectioning system (Warner Instruments, Hamden, CT, USA). Viable sections were then transferred back to media in glass bottom culture plates and imaged immediately on an inverted confocal LSM710 microscope (Zeiss, Welwyn Garden City, UK). After imaging, organoids were dissociated on the following day for flow analysis.

### Imaging

Imaging of cell cultures and tumour organoids was performed on a laser scanning microscope (LSM) 710 (Zeiss, Welwyn Garden City, UK) using separate tracks for t-sapphire and Venus and a combined track for EBFP2 and mOrange2 fluorophores. The details of the fluorochromes, excitation and emission maxim are, laser wavelength and bandpass filters are depicted in Fig. 1B and listed in Supplementary Table 2. T-sapphire is a brighter fluorophore than EBFP2, which is accounted for by a higher 405 nm laser power for EBFP2 collection and a lower power for T-sapphire collection. Venus was excited by 488 nm to avoid bleed through from mOrange2. For acquisition of images from P5 cultures and colony formation, linear unmixing and online fingerprinting were used (Zeiss, ZEN software) for more accurate separation of overlapping signals. Reference spectra were recorded by imaging a population expressing a single fluorophore (EBFP2, T-sapphire, Venus, or mOrange2) with simultaneous exposure with 405, 488, and 561 nm laser lines and appropriate gain. Regions of interest corresponding to individual fluorophore were manually selected to achieve in-software reference spectra, which were used to separate fluorophore signals in real time through online fingerprinting.

### Data analysis

Surface-marker-based phenotypes were determined by binarizing signal intensities for each surface marker on each cell as positive or negative. Flow cytometry gating to determine positive and negative cut offs were manually drawn based on profiles of singly labelled controls (Supplementary Fig. 4). Thus, 16 combinations (surface marker profiles, SMP) are possible from 4 surface markers (Fig. 3A). The relative proportions of the 16 combinations within a subset of cells (cell line/whole culture/clone) are regarded as representing a phenotype. Estimations of the degree of similarity of any two phenotypes were determined using the Cosine of Similarity. Therein, the similarity of phenotypes for any two subsets of cells was determined as the inner product of their 16-dimensional vectors. When the Cosine of Similarity is 1 the samples have the same phenotype and from 1 to 0 are increasingly dissimilar. Assessments of the similarity of more than two subsets have been achieved by determining the cosine of similarity of all pair-wise combinations.

### Statistical analysis

To aid interpretation of the Cosine of Similarity metric, we undertook a Monte Carlo simulation by repeatedly sub-sampling an in-silico population (size based on empirical batch data) with a hypothetical phenotype comprising 16 different proportions of surface marker profiles. Repeatedly choosing (×100,000) sub-samples of cells (i.e., of the order empirically seen for single clones) without replacement enabled probability distribution curves for expected deviations in similarity between the phenotype of the population and the sub-samples. Under such conditions, the lower values of CoS are reported for the experimental conditions that were observed with a probability of *p* < 0.001 (black dashed lines diagrams).

## Results

### Combination of 4 fluorophores generates 6 dual-labelled (barcoded) populations

We used lentivirus-mediated expression of fluorophores to barcode-label cells. Mixtures of four lentiviruses expressing EBFP2 (B), T-Sapphire (S), Venus (V), and mOrange2 (O) (Fig. 1A) were used to transduce GIC. Six separate dual transductions were performed, with approximately equal amounts of viral particles. The colours of the lentivirus-expressed fluorescent proteins were chosen to leave a considerable portion of the red and far-red spectrum to allow adjunct fluorescent surface marker labelling (Fig. 1B). We observed formation of clusters on flow cytometry dot plots, which have been described previously^26,43^ and are considered clonal expansions. Two cell lines derived from IDH-wildtype glioblastomas, designated G19 and G61, were used.

Flow cytometry of passaged cells expressing two fluorophores shows discernible “streaks” or “clouds” indicative of clonal populations that are not easily discernible in clones expressing a single fluorophore^28,43^. The generation of triple- or quadruple-label cell populations, to expand the number of distinctly labelled clonal populations have been proposed previously^28^ but we found that triple labelled GIC had a growth disadvantage compared with single- and dual-labelled cells. Therefore, the generation of dual-label barcoded cells only was considered as a practical compromise to eliminate impact of label expression on cell growth whilst permitting accurate detection of clonal populations in a culture. This resulted in dual-labelled cultures carrying the fluorophore combinations (barcodes) BS, BV, BO, SV, SO, and VO (Fig. 1C) in approximately equal distributions (Fig. 1D), similar to previous reports^28^. The variability of label groups was ascribed to inaccuracies in viral titre estimates leading to a modest predominance of certain label groups (Fig. 1D). Figure 1E outlines the experimental workflow of this study, with individual experimental steps (ES 1–ES 7) indicated for further reference. In vitro manipulation, in particular the viral barcoding approach can have an impact on cell viability and self-renewal capacity. We have performed clonality assays and show that the viability and the self-renewal capacity of naïve, non-barcoded GIC is broadly similar that of double-barcoded GIC (Supplementary Fig. 3).

### Generation of multiple distinct clones in culture identified by barcode labelling

**Fig. 2:**
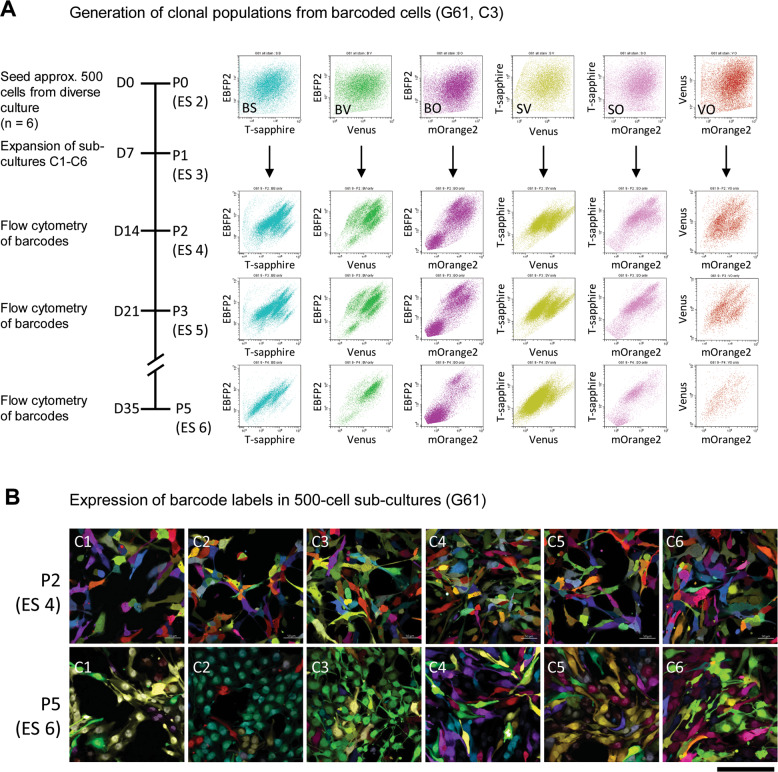
Clonal populations emerge during passaging of barcode-labelled cells. **A** Flow cytometry of barcode-labelled cells (G61) before seeding at P0, (ES 2). Expansions of cells into subcultures C1–C6 and formation of clonal population visible as “clouds” or “streaks” during propagation. Clonal formation in culture C3 (ES 2–ES 6), corresponding to passages P2, P3, and P5. **B** Confocal imaging of cultures C1-C6 at P2 and P5, showing formation of emerging clonal populations of barcoded cells (ES 4, 6). Scale bar corresponds to 50 µm.

Subcultures were prepared by seeding 500 barcoded cells of G19 and G61. In our settings, the propagation of such cultures reliably produces “streaks” in FACS plots of dual-labelled cells that could easily be delineated and followed across passages (Fig. 1C, E, ES 4). Flow cytometry was performed on barcoded populations when seeding cells into individual (sub)cultures C1-C6 (ES 3) and at passages P2, P3, and P5, corresponding to ES 4-ES 6. Clones were detected after 14 days at P2, identified as well-demarcated streaks on flow cytometry plots in cultures G61 (Fig. 2A) and G19 (Supplementary Fig. 5A). Over the passages these streaks remained at the same positions on flow diagrams, but showed fluctuations in size, depending on the cell number constituting these individual clones. With this approach, we were able to produce a number of traceable clones within 2 passages (2 weeks), without requiring single-cell cloning, expansion and maintenance as previously described^28^. Representative images of 500-cell subcultures C1-C6 at P2 (ES 4) and P5 (ES 6) are shown in Fig. 2B (G61) and Supplementary Fig. 5B (G19). Clonal expansions show as predominance of a smaller number of distinct labels in the populations at P5, which also leads to a decrease of the overall number of clones over passages. In conclusion, purified dual-labelled glioma cells are capable of forming clonal populations which emerged after as little as two passages from mixed cultures.

### Surface marker phenotyping in mixed barcoded cells during parallel propagation reveals diverse properties of distinct glioma lines

**Fig. 3:**
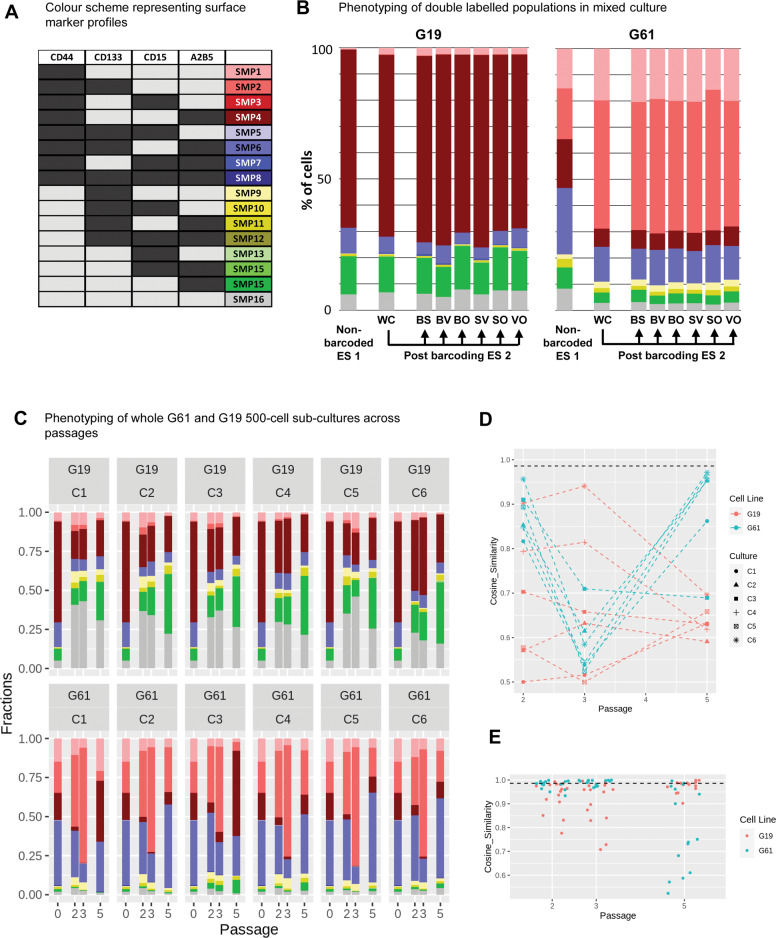
Surface marker expression on whole cultures and barcoded subpopulations. **A** Colour scheme to identify combinations of surface marker expression of CD44, CD133, CD15, and A2B5, similar to ref. ^24^. **B** Surface marker expression (“phenotyping”) of non-barcoded G19 or G61 glioma cells (ES 1), compared to the pool of bar-coded cells (post barcoding, WC [whole culture]; ES 2) and separate phenotypic analysis of the individual bar-coded cells (post-barcoding, clones BS, BV, BO, SV, SO, VO). The phenotype of G19 cells prior and post barcoding undergoes less changes than that of G61 where a shift towards CD44+, CD133+ dominance is observed. **C** Phenotyping of subcultures C1–C6 of cell lines C19 and C61. Each graph shows at the left the column for P0 (post-barcoding, ES 2), and subsequent passages P2, P3, P5 with phenotypes for the whole population in each culture (C1-C6). **D** Plot of cosine of similarities of cell lines G19 and G61 at different passages compared to P0 (ES 2). Cell line G19 starts with a low degree of similarity between cultures C1-C6, with a range of very similar to very dissimilar cultures (compared to P0; *Y* axis, cosine of similarity) whilst the degree of similarity is more consistent in culture G61, starting with a higher degree of similarity between cultures compared to P0, which reduces at P3 and gains at P5, i.e. G61 vary over passages but the cultures C1–C6 are coherent with each other. The loss of coherence with P0 at P3 recovers by P5. **E** Assessment across cultures C1–C6 per passage and culture (C19, C61). Each point is a comparison of two cultures. G19 and G61 exhibit different profiles, with G61 showing coherence within P2 and P3, with a loss of coherence at P5 and G19 showing lack of coherence within P2 and P3, but regain at P5. Black dashed line in **D** and **E**—below line *p* < 0.001 (based on Monte Carlo simulations).

The plasticity of bulk glioma stem cell cultures has been previously demonstrated, highlighting a continued flux of non-hierarchical, reversible surface marker states^24^. Here we examined how SMP associate with barcoded GIC clones present in subcultures of G61 and G19 (Supplementary Fig. 6). First, we analysed the SMP of the two IDH-wildtype GIC lines, designated G61 and G19, prior to barcoding. Cells were surface labelled with antibodies against CD44 (APC-780), CD133 (BV711), CD15 (BV780) and A2B5 (APC) to identify combinations of 16 distinct SMP (designated SMP1-SMP16, and correspondingly colour coded, Fig. 3A). In keeping with previous reports^24^, we find that the cell lines (G19 and G61) exhibit distinct relative proportions of the SMP (Fig. 3B). With CD44 expression dominant but variably associated with A2B5 (G19) or CD133 (G61) illustrates the unique phenotypes as defined by relative proportions of SMP associated with each cell line in a naïve bulk population preparations (Fig. 3B, first stacked column; “non-barcoded ES 1”).

Surface marker antibody fluorophores were selected for excitation and emission spectra separate from the barcode labels (Fig. 1B). Combination of the two systems minimally altered the phenotype of the whole culture for cell line G19, whereas there was a subtle change in G61 (Fig. 3B). Post-barcoding, G61 shows a reduction of the A2B5 component in the triple-positive phenotype CD44+, CD133+ and A2B5+. Individual barcodes (BS, BV, PO, SV, SO, and VO) within each cell line exhibited similar phenotypes to the whole culture, with a cosine similarity of all barcoded populations relative to the barcoded whole culture >0.99. Supplementary Figs. 7 and 8 show the flow cytometry plots of the forming clonal populations and the proportional contribution and dynamics across passages.

Six cultures (C1–C6) were seeded from the same bulk population (P0) of each cell line and their cell SMP-associated phenotypes evaluated over subsequent passages P2, P3, and P5 (Fig. 3C). Objective quantification of the similarities of phenotypes for each culture, C1-C6, across passages, P2–P5, relative to passage P0 (Fig. 3D) and within passage (Fig. 3E) revealed the dynamics associated with cell lines G19 and G61. Strikingly, G19 and G61 showed distinct differences in their similarity to P0 over subsequent passages. G19 starts (at P2) with a broad range of degrees of similarities for cultures C1–C6, compared to P0, i.e., with values ranging from 0.5–0.9, which is maintained in P3, and at P5 all cultures (C1–C6) converge to degrees of similarity between 0.5–0.7 (Fig. 3D). Indeed, the coherence of the independent cultures of G19 at P5 is confirmed in the pair-wise comparisons with values >0.9 (Fig. 3E). In contrast, G61 cultures C1–C6 have initially a high degree of similarity to P0 with values between 0.8 and 0.95. This degree of similarity drops significantly at passage 3 (CoS 0.53–0.71) and regains at passage 5, where 4 of the 6 cultures return to a high degree of similarity to the seeding population (CoS > 0.95). This lack of coherence in G61 at P5 is highlighted in the range of values associated with the pair-wise comparisons between cultures (Fig. 3E). Overall, the across passage comparisons revealed highly dynamic and diverse changes in phenotypes that were not coherent across cell lines. Broadly, G19 seeded diverse cultures that became more coherent over time, whereas G61 seeded coherent cultures that became less coherent over time.

In conclusion, computational analysis of the distinct subcultures (C1–C6) in comparison to P0 reveals distinct patterns of divergent surface marker evolution between two cell lines, and also distinct patterns of surface marker expression when comparing individual subcultures (Fig. 3D). Importantly, at this stage, the phenotypic analysis was performed on all cells in the subcultures without considering barcode labels.

### Analysis of coherence of clones reveals phenotypic shifts across cultures and passages

Here we assessed how the phenotypic shift of cell populations between cultures and during passages might be driven (i) by dynamic shifts in phenotype of one clone, (ii) of many clones, or (iii) cannot be attributed to changes in the delineated clone. To this end, we delineated clonal populations by manually gating and matching across passages P2, P3, and P5 (examples in Supplementary Fig. 6). In total, we extracted data for 104 and 84 clones across all cultures C1-C6 for G19 and G61, respectively, and determined clonal phenotypes (Supplementary Fig. 9).

**Fig. 4:**
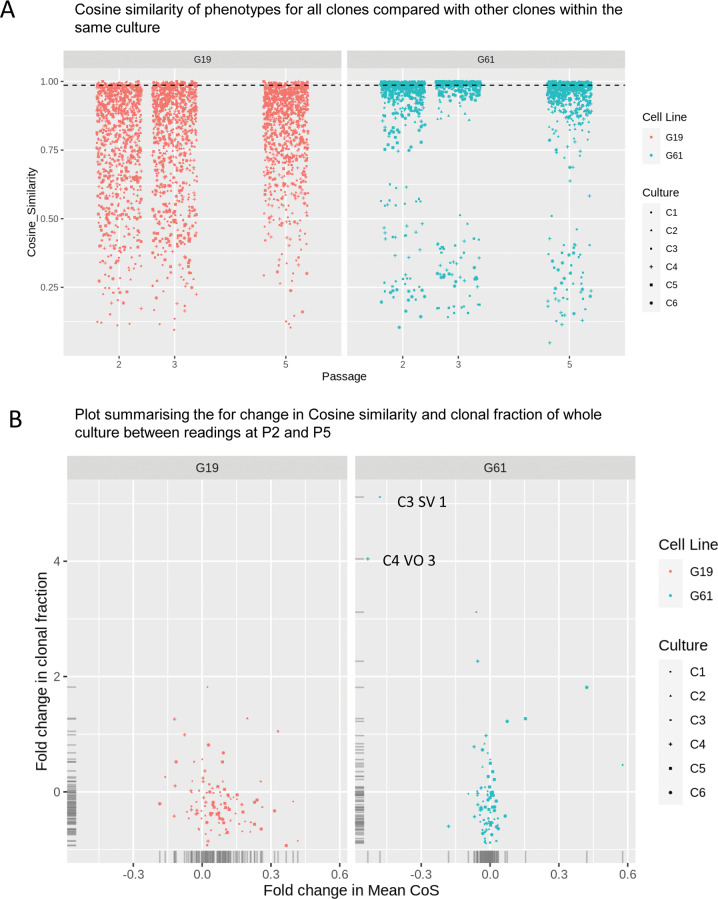
analysis of similarities of surface marker expressions of clonal populations. **A** Assessment of similarity of whole cultures with each subculture C1–C6, and passages P2-P5. The analysis indicates a high diversity of phenotypes across clones (BS, BV, PO, SV, SO, VO) in G19 compared to G61. The similarity of clones in the G19 culture is continuous, whereas G61 appears bimodal. **B** Display of fold changes in coherence and fraction of clone size to the whole cultures. G19 cells show a greater spread of the fold changes of cosine of similarity (*x*-axis), indicative of less coherent phenotypes, but there is also less variability in clonal sizes (*y*-axis). G61 cells show a higher degree of coherence (less spread on the x-axis) but also a much higher dispersion of clone size with some clones showing significant size changes. Annotated data points (C3 SV 1 and C4 VO 3) are represented in Supplementary Fig. 11 and visualisation of clonal expansion in Fig. 2.

Exploring the coherence of clones within each passage, we determined the cosine of similarity for all pairs of clones identified within each culture (C1-C6), i.e. for *n* clones in a culture there would be *n* × (*n* − 1)/2 pair-wise comparisons. The resultant distributions of values for the cosine of similarity are strikingly different for the cell two cell lines (Fig. 4A, Supplementary Fig. 10A). First, the extent of dissimilarity between the clones of the G19 cell line is much greater at all passages and within each culture than the clones of the G61 cell line. Indeed, the dissimilarity between clones of the G19 cell line is much greater than dissimilarity between the subcultures (Fig. 3E) suggesting a high diversity of phenotypes within the clones may explain the lack of coherence of between subcultures at P0 and P2. Interestingly, the range of cosine of similarity values for paired clones decreases by P5 which is consistent with the coherence of the whole culture phenotypes (Fig. 3D, E). As the gated clones comprised 55–87% of the whole population within each culture (see methods section) it is reasonable to assume the changes in phenotype at the whole culture level can be attributed to the dynamics of the clonal phenotypes. In contrast, the clonal level pair-wise comparisons for G61 are more bimodal with the majority of clones being highly similar to each other with a subset of clones showing highly dissimilar phenotypes. This is visualised in Fig. 4B (G61) and in the boxed areas of Supplementary Fig. 9B, where outliers appear dissimilar to the other clones in the G61 culture. To explore the dataset further, the average of all paired comparisons for each clone was determined and the fold change between P2 and P5 calculated. Plotting against the fold change in clonal fraction (ratio of the number of cells in the clone to the number in the whole sample) for each clone facilitated an exploration of the influences of the identified clones on the whole culture (Fig. 4B).

For both cultures, the skew to negative fold change in clonal fraction for both cell lines suggests the majority of identified clones are decreasing in their contribution, for example, due to the emergence of cells in other clones, reflected in a re-formation of clouds. For fold changes in average similarity with other clones in the culture, the skew to the right of cell line G19 suggests the culture is becoming more coherent at the clonal level, while the G61 cell line appears very stable, with occasional exceptions (annotated data points C3 SV 1 and C4 VO in Fig. 4B and visualised in the corresponding annotation in Supplementary Fig. 9), which are in keeping with the bimodal distribution in Fig. 4A. The same analysis was performed for individual barcoded doublets (BO, BS, BV, SO, SV, VO) across cultures (C1–C6), for cell lines G19 and G61. A detailed view of the similarities of these individual barcode-doublets is shown in Supplementary Fig. 10B (overview) and 10C (detailed view), for cultures C19 and C61.

In conclusion, the analysis of the coherence of the phenotypes of clones reveals distinct behaviour between cell lines and across clones, cultures and passages.

### Microenvironment modifies spatial organisation of clonal populations in tumour organoids

**Fig. 5:**
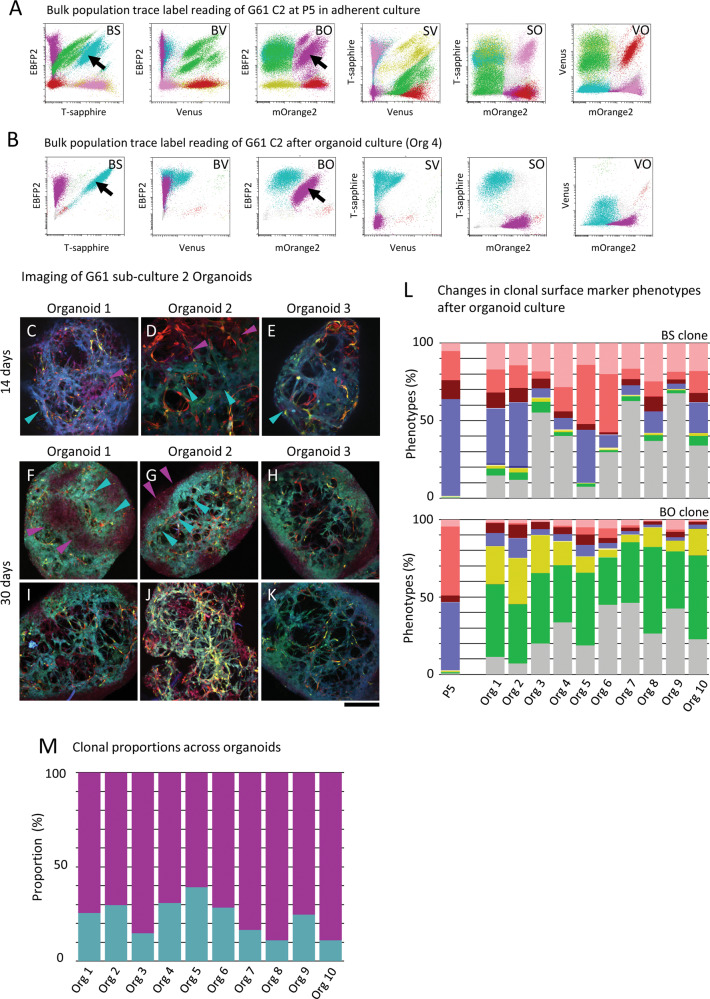
Maintenance of clonal predominance and plasticity of surface marker expression in tumour organoids. **A** P5 culture with a predominance of clonal populations with barcode label BS and BO (cell line G61) to generate organoids. **B** Flow cytometry of dissociated organoids show the two dominant label populations BS and BO. Arrowheads indicate clonal populations which continue their dominance in organoids. **C**–**E** Three separate organoids grown from cells with colour barcodes BS and BO, 14 days after seeding, showing both populations of barcoded cells populating the organoid in specially organised patterns. Arrowheads in **C**–**E** point to populations of barcoded clones. Three separate organoids (surface aspect) (**F**–**H**), and the same organoids centrally cross-sectioned (**I**–**K**) showing spatially organised BS and BO clones as indicated by arrowheads in **F** and **G**. **L** These label populations were then analysed for surface marker expression showing A2B5-dominant populations overall, whilst separate analysis of label barcoded clones shows surface marker separation when analysing clones BS and PO separately, with a marked difference of A2B5 expression, and a notable dominance of cells negative for all surface marker in the BS clone. Colour codes see Fig. 3 and Supplementary Table 3. **M** Proportion of barcoded clonal populations across ten separately analysed organoids. Scale bar in **C**–**K** corresponds to 500 µm.

To understand how barcode-labelled cell populations might grow under changing selection pressure in different culture conditions, we prepared organoid cultures (tumour spheres) with traceable clonal mixtures. Figure 1E illustrates the organoid preparation in the experimental workflow. Organoids were derived from cells at two different experimental steps from barcoded GIC - “oligoclonal” organoids derived from P5 cultures which already contain dominant barcoded clones (at stage ES 6) and from “polyclonal” cultures directly after barcoding (ES 3) but prior seeding into, and passaging of, subcultures. At ES 3, whole P5 populations, containing dominant clones (e.g., G61 BS and BO; Fig. 5A), were used to generate organoids. Here, analysis of the surface markers of the whole P5 culture (Fig. 3C) shows a distribution that is broadly similar to that in the subclones BS and BO (Fig. 5L), in this instance dominated by CD44+ CD133+, A2B5+ expression. The forming organoids maintained the dominance of presumed clonal populations with barcodes BS and BO. These populations showed incipient formation of barcode label foci at 14 days (Fig. 5C–E), and a more accentuated clustering emerging after further two weeks of organoid culture (Fig. 5F–K). The analysis of individual organoids after 30 days in culture by flow cytometry confirms the presence of the same distinct clones with barcodes BS and BO (Fig. 5B). However, the organoid culture conditions have resulted in a significant shift of the surface marker expression in the pooled population with a dominant expression of A2B5 (green), and a population expressing none of the four surface markers (grey) (Fig. 5L). Strikingly, the separate analysis of the two clonal populations shows a marked difference of phenotypes between clones BS and BO, where the more superficially located BO clone (magenta, Fig. 5G) is strongly dominated by A2B5+ cells (Fig. 5L, lower panel), whilst cells populating the core were mostly comprised of BS clones which had a higher proportion cells with SMP16 (i.e. no expression of the 4 surface markers, Fig. 3A), but also showed an expansion of CD44+ cells with variable additional expression of A2B5 and CD133 (Fig. 5G, L, upper panel). This suggests the regional expansion of clonal populations, and this is accompanied by plasticity of the surface marker phenotype. In a subset of experiments, we observed an emergence of single-label clones which had not been entirely eliminated from the starting culture (Supplementary Fig. 11). Here, the double-label clone with barcode SV, but also an additional single-label clone (O) constituted the organoids, and there is a surprising dominance of the single-label clone. This spatial organisation into clusters in G61 however is contrasted by a much more dispersed growth and an absence of cluster formation in organoids derived from G19 P5 cultures (Supplementary Fig. 13A). Flow cytometry analysis of 13 such G19 organoids (day 30) from cultures C1 (*n* = 4), C2 (*n* = 3), and C3 (*n* = 6), (Supplementary Fig. 13) shows the presence of multiple clones in each organoid. A proportion of the organoids harboured multiple clones of the same barcode colour (Supplementary Fig. 12C, BV barcode, organoid 2). The SMP showed variation across different organoids, but also across distinct barcoded populations (Supplementary Fig. 13B).

Finally, we analysed the capacity of barcoded, polyclonal populations (ES 3, prior to seeding into subcultures) to form populations of spatially segregated barcode labels (ES 7) (Supplementary Fig. 14). G19 cells show in a proportion of organoids a tendency to regional enrichment of barcoded populations (Supplementary Fig. 14A #2,#3, #6, #9), however, the majority of organoids shows a predominantly diffusely disseminated distribution of the different barcode populations (Supplementary Fig. 14A, #10–18). Instead, G61 organoids grown from polyclonal populations show a higher propensity to form spatial arrangements of barcoded cells (Supplementary Fig. 14B, #1–12).

In conclusion, the changing microenvironment, generated by transferring clonal populations from adherent culture into organoid conditions, had a profound impact on the adjustment of the phenotypes of distinct clones, with a differential preference of phenotypes related to spatial location in the organoid, in particular in G61. We also found that the two cell lines G19 and G61 develop distinct patterns in spatial reorganisation in organoids, both, as oligoclonal population (Fig. 5, Supplementary Fig. 11) and as polyclonal population (Supplementary Fig. 14), suggesting distinct inherent responses to changes in microenvironment.

## Discussion

The genetic and functional intratumoural heterogeneity of cancer cells remain a complex and difficult-to-model aspect of tumour biology. There are multiple powerful modifiers of CSC states, including genetic heterogeneity, environmental (inflammasome, stroma), and metabolic factors, such as hypoxia, glucose concentration, and pH levels. The inherent plasticity of CSCs in adapting to these environmental cues renders them a diverse and dynamic target for therapeutic intervention^24^, i.e., there are potential escape mechanisms from therapies that aim at populations of a certain differentiation state. To understand tumour evolution, it is important to follow the fate of individual cancer cells and their contribution to the bulk tumour. Through this approach, seminal studies have demonstrated clonal hierarchies in glioblastoma^35,44^ and other cancers^45,46^. However, at the same time tumours show a remarkably consistent spatial epigenetic profile^47^, suggesting that genetic background only partially explains the variety of cellular phenotypes observed in a single tumour. To address evolutionary aspects of CSCs, multiple tracing and labelling techniques have been developed^28,29,33^. These are powerful techniques for investigating clonal dynamics but are limited in their ability to capture plasticity and microenvironmental interactions. An important contribution to the understanding of clonal evolution of barcoded glioblastoma cells came from serial xenotransplantation studies, which concluded that clonal expansion of glioblastoma GIC involves a conserved proliferative hierarchy^35^. Here we set out to create a model system that is informative of both clonal dynamics and phenotypic plasticity, through barcoding and clonal selection and concomitant phenotyping with GIC surface markers. The approach to enrich for clonal populations could represent an experimental advantage over approaches using a far more complex system that may involve single-cell cloning, simultaneous maintenance of established clones, and xenografting. It also eliminates the additional variable of a microenvironment including mesenchymal and inflammatory cells. Simultaneous clonal tracking and marker phenotyping was accomplished by a reduction in the complexity of the barcoding system^28^ to a combination of four fluorophores, yielding combinations of six dual-labelled (barcoded) cells. We found that single-label cells had the potential to outgrow dual-labelled populations, which were interpreted as a growth advantage, possibly due to single-label-only expression. We therefore removed single labels by FACS sorting at ES 2. In keeping with previous reports^43^, following serial passaging, we observed the formation of clonal populations, visualised by the appearance of “clusters” or “streaks” on flow cytometry dot plots (Figs. 2A and 5A, Supplementary Figs. 5A, 6, 9A, 10). Not unexpectedly, and in keeping with previous studies^24,35,48^, there was considerable phenotypic diversity between the two cell lines (Fig. 3C, D) but interestingly we also observed diversity between serially traced barcoded clonal populations of the same cell line (Fig. 4A, B, Supplementary Fig. B, C). Clonal diversity was quantified by computational analysis of phenotypes within cultures (Fig. 4A, B), and across barcoded populations (Fig. 4C, Supplementary Figs. 9, 10). The transfer of P5 passaged barcoded glioma stem cells of line G61 containing dominant barcoded populations (Fig. 5A, BS, BO) into a different microenvironment by generating tumour spheres (organoids) showed not only a remarkable persistence of these dominant clones (Fig. 5B) but also at organoid level a striking spatial organisation evolving during organoid formation (Fig. 5C–K). Compellingly, cells derived from line G19, whilst also forming dominant barcoded populations, showed a much lesser degree of spatial organisation in organoids, despite persistence of dominant barcode label populations.

In keeping, such behaviour of G19 and G61 barcode-labelled cells was also maintained when cells were seeded prior to formation of distinct barcoded clonal populations (ES 3 & ES 7) (Fig. 1E, “pre-clonal” population, seeded directly to form “mixed” organoids). Mixed organoids derived from G61 cells show regional distribution patterns that are different from those formed from G19 cells (Supplementary Fig. 13A, B), i.e., there is formation of either a disseminated (G19, Supplementary Fig. 14A) or a clustered (G61, Supplementary Fig. 14B) pattern, similar to populations seeded from P5 subcultures (G19, Supplementary Fig. N9, and G61, Fig. 5 and Supplementary Fig. 11). The formation of such distinct patterns suggests inherent, and surprisingly stable properties of GIC, resembling the observations from more complex glioma stem cell models^35^. In our experimental setting, we observed (i) selective propagation of dominant barcode label populations (Fig. 5M), (ii) formation of distinct, cell line-dependent, spatial organisational patterns of these clones (Fig. 5C–K, Supplementary Figs. 11 and 12) and (iii) divergence of SMP phenotypes of the various barcoded populations (Fig. 5L, Supplementary Figs. 11 and 12), suggesting that barcoded populations can adapt their phenotypes in response to changing environmental cues. A possible explanation for such diversity could be a difference in nutrient availability and oxygen pressure between the outer and inner parts of the sphere, and a different response to extracellular matrix.

Whilst our study explores important aspects of relationships of clonal populations, identified by barcode labelling, to stem cell phenotypes, there are limitations that will require further exploration. First, this study is based on two glioblastoma stem cell lines, with slightly different genetic profiles and related, though distinct methylation subclasses. Therefore, future studies will need to include additional glioblastoma cell lines and will need exploring the dynamics of clonal populations over further serial passages, and potentially an exposure to additional modifications to the environment. We mitigated experimental variables by performing the entire series of experiments strictly in parallel using equal culture conditions, but undoubtedly additional, massive parallel cultures will yield a more comprehensive dataset for computational analysis. A general limitation of this and other studies is the choice and availability of stem cell markers to characterise GIC. However, the main purpose of surface marker profiling in this study is to demonstrate plasticity of clonal populations, across passages and during organoid formation and therefore this limitation is of minor relevance only. A further limitation is the propagation of GIC and the culture of organoid in a context lacking interaction with a microenvironment such as stromal cells, endothelial cells, macrophages, which can further modulate SMP. Whilst these extrinsic modifiers have been omitted, our model however provides in the first instance a picture how even comparatively well-controlled culture conditions can modulate marker expression in GIC. To define the contribution to GIC SMP, further experiments with additional extrinsic components (endothelial cells, fibroblasts, macrophages) can help dissecting the role in phenotypic modulation. We also observed a potentially generic limitation of the barcoding system whereby single-label cells can dominate a culture (Supplementary Fig. 11), whereas triple labelled cells had a growth disadvantage. This imbalance was mitigated by eliminating single-label cells.

In conclusion, we present here an experimental approach to barcode glioma-initiating cells and create clonal populations, which can be phenotyped simultaneously. Through computational analysis, we are able to pinpoint the fate of such populations, which can be used to interrogate the phenotypic plasticity in response to changing tumour environment. The simplification of the workflow of clonal selection, combined with reproducible and robust readout to assess the functional properties of GIC renders this assay potentially very suitable for screening of newly established GIC for tumour-specific therapeutic vulnerabilities and to assess the impact of experimental genetic or epigenetic modifications.

## Supplementary information

Supplementary Material

Supplemental data 1

Supplemental data 2

Supplemental data 3

Supplemental data 4

## Data Availability

The datasets used or analysed during the current study are available from the corresponding author on reasonable request.
