# Supplementary material for: Phenotyping clonal populations of glioma stem cell reveals a high degree of plasticity in response to changes of microenvironment
Source: Lab Invest. 2021 Nov 15;102(2):172–84. doi: 10.1038/s41374-021-00695-2 (PMC8784315; doi:10.1038/s41374-021-00695-2)
Supplement: Supplementary file 1 — Supplementary Material [file 41374_2021_695_MOESM1_ESM.pdf]

Supplementary figure 1

Copy number profiles of primary tumours

A G19 (NH15-2101)

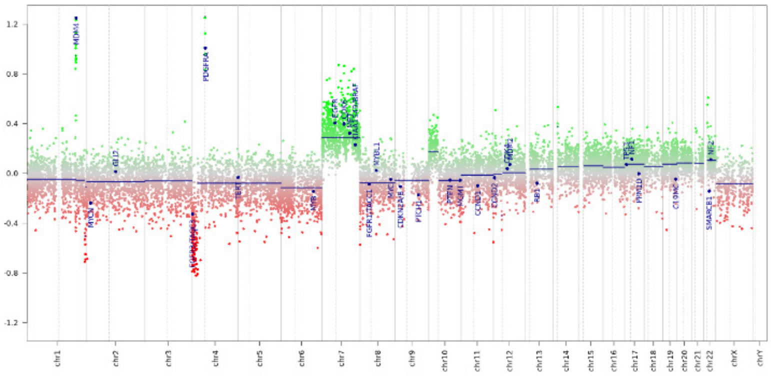

B G61 (NH16-2806)

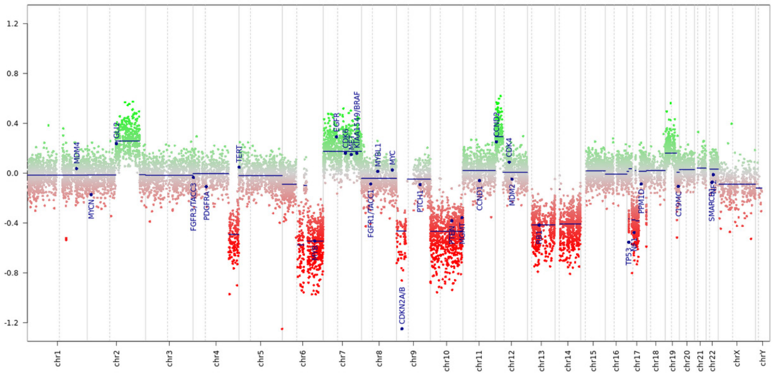

Supplementary Fig. 1: copy number profile of the primary tumours from which cell lines G19 and G61 were derived. A, copy number profile of the IDH-wildtype glioblastoma from which cell line G 19 was derived. There is a striking MDM4 and PDGFR amplification, as well as a characteristic gain of chromosome 7. B, copy number profile of the parent tumour corresponding to G61: multiple copy number gains and losses, with a characteristic chromosome 7 gain and chromosome 10 loss, and in addition a CDKN2A/B deletion, which is a feature of a proportion of glioblastomas.

Supplementary figure 2

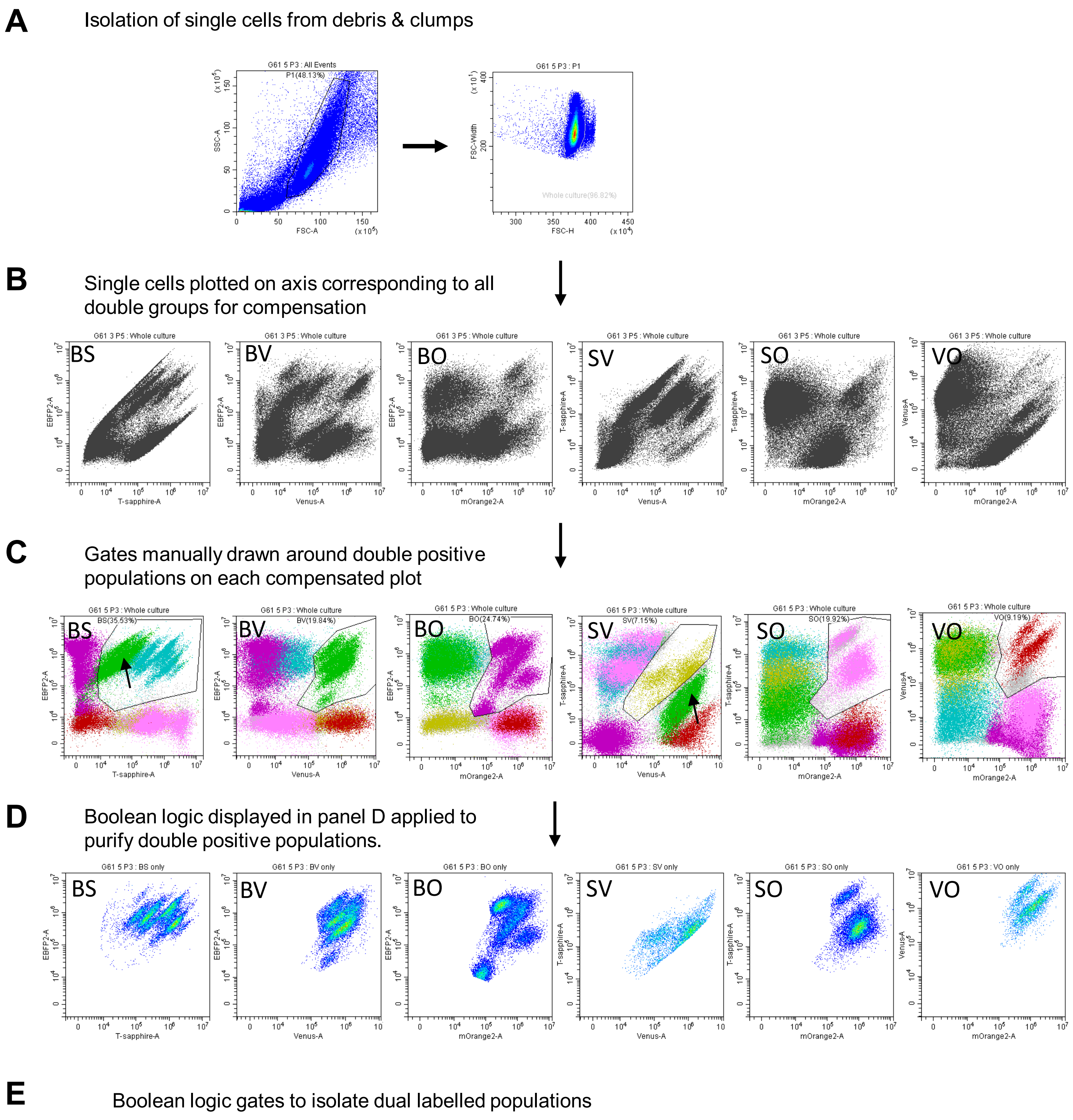

Supplementary Fig. 2: Gating to isolate cell detections corresponding to double labelled cells. A, side scatter area (SSC-A) and forward scatter area (FSC-A) were used to remove dead cells and debris in the preparation. This P1 population was then plotted on FSC-height (H) and FSC-width (W) to remove any possible doublet cells. **B,** Purified cell detections were then plotted on flow plot axis permitting detection of each of the six double positive populations (BS, BV, BO, SV, SO, VO) and compensation applied to the cells. Pilot experiments using single colour control cells (B, S, V, O) were performed to determine sufficient separation of fluorescent signals. **C**, After compensation, gates were drawn to isolate the dual labelled populations that contain clonal streaks we aim to trace. Arrows in BS and SV plots show that even after compensation, the double positive population BV (green) bleeds through considerably into the t-sapphire channel. This is caused by Fluorescence Resonance Energy Transfer (FRET) whereby 405 laser exciting t-sapphire will also excite EBFP2 in the BV population. EBFP2 emission in turn excites Venus which can also be detected in the 525/40 nm filter used for T-sapphire. Effects of this on accurate detection of double labelled populations have been mitigated by stringent gating and separation through Boolean logic gates D. Finalised double positive populations achieved through Boolean logic to remove any events that are detected across more than one double positive gate. This population is used to pull events belonging to streaks/clusters. D. Boolean logic gates to achieve final populations for tracing. Note, for BV the gate “not BS” is omitted because of FRET described earlier.

Supplementary figure 3

A Gating for dual label single cell sorting

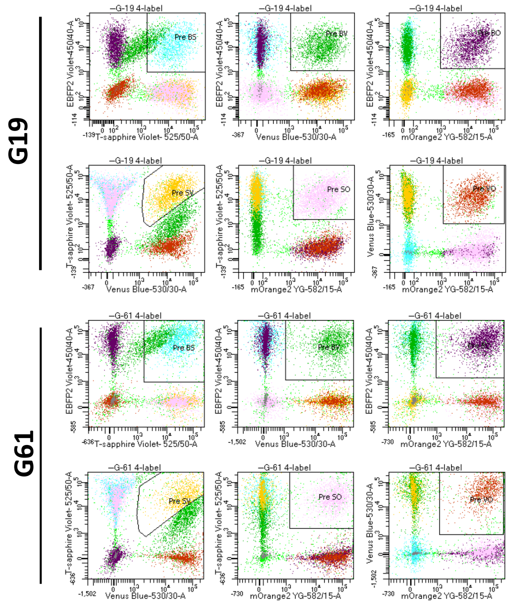

B Quantification of single cell growth

	Naïve	Unlabelled	BS	BV	BO	SV	SO	VO
G19	21	18	30	15	13	16	17	19
G61	15	22	3	24	20	18	20	21

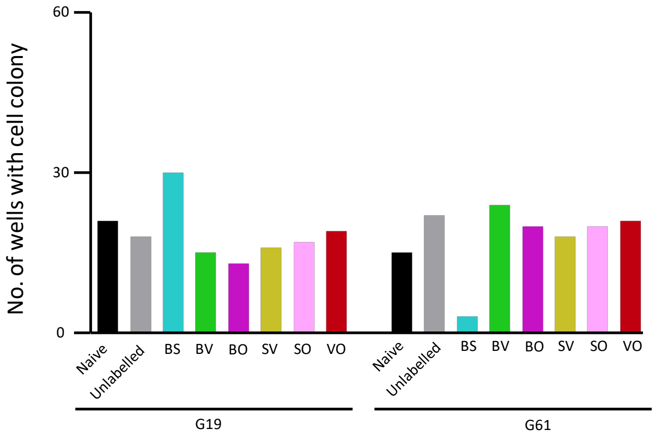

C **G19 clones (20x objective)**

D **G61 clones (10x objective)**

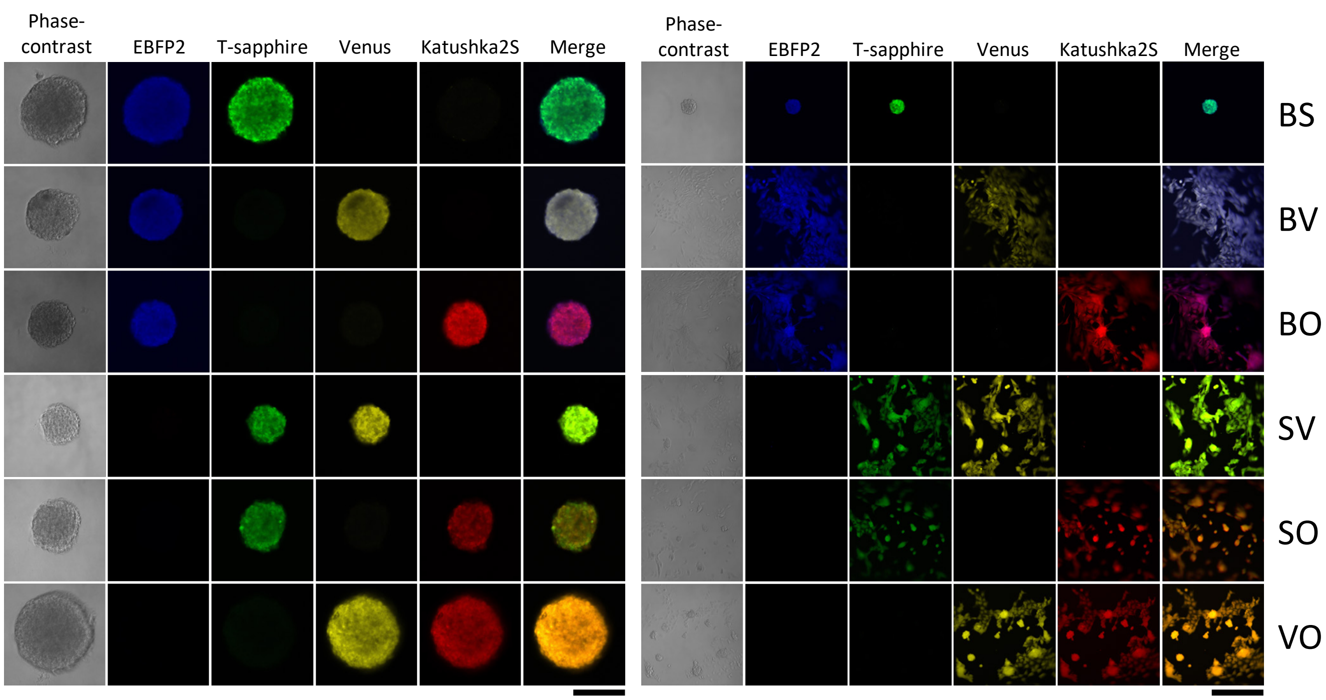

Supplementary Fig. 3: Single-cell FACS sorting and self-renewal of naïve and double labelled G19 and G61 populations. **A** FACS gating for single-cell sorting of all six dual-labelled populations in G61 and G19. Single cells were sorted into the 60 internal wells of a 96-well plate. One plate each for unlabelled, naïve GIC, for unlabelled virally exposed cells and for each dual-label group (BS, BV, BO, SV, SO VO). **B**, Quantification of single-cell colony formation for each of the different sorted populations. N = 60 single cells sorted per condition. C, D, Linear unmixed confocal imaging of representative single cell colony formation for G19 or G61. Scale bar corresponds to 100 µm (C) and 200 µm (D).

Supplementary figure 4

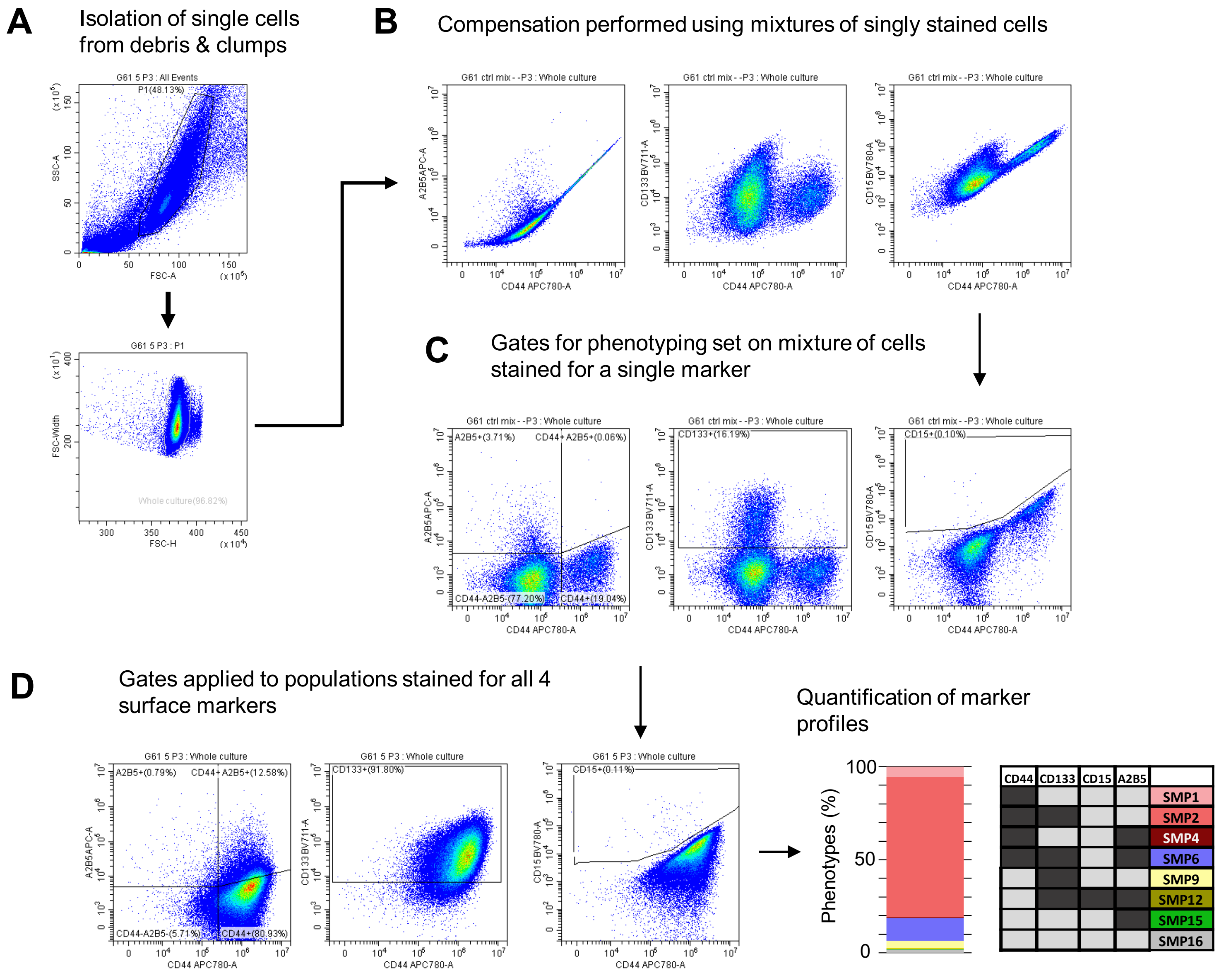

E Boolean logic to isolate 16 different phenotypic populations applied to samples stained for all 4 fluorophores

CD44+ only =	NOT(CD44+ A2B5+) AND NOT(A2B5+) AND NOT(CD44-A2B5-) AND CD44+ AND NOT(CD133+) AND NOT(CD15+)
CD44+ CD133+ =	NOT(CD44+ A2B5+) AND NOT(A2B5+) AND NOT(CD44-A2B5-) AND CD44+ AND CD133+ AND NOT(CD15+)
CD44+ CD15+ =	NOT(CD44+ A2B5+) AND NOT(A2B5+) AND NOT(CD44-A2BS-) AND CD44+ AND NOT(CD133+) AND CD15+
CD44+ A2B5+ =	NOT(A2B5+) AND CD44+ A2B5+ AND NOT(CD44-A2B5-) AND NOT(CD44+) AND NOT(CD133+) AND NOT(CD15+)
CD44+ CD133+ CD15+ =	NOT(CD44+ A2B5+) AND NOT(A2B5+) AND NOT(CD44-A2B5-) AND CD44+ AND CD133+ AND CD15+
CD44+ CD133+ A2B5+CD44+ =	A2B5+ AND NOT(A2B5+) AND NOT(CD44-A2B5-) AND NOT(CD44+) AND CD133+ AND NOT(CD15+)
CD44+ CD15+ A2B5+ =	CD15+ AND NOT(CD133+) AND NOT(CD44-A2B5-) AND NOT(A2B5+) AND CD44+ A2B5+ AND NOT(CD44+)
CD44+ CD133+ CD15+ A2B5+ =	NOT(CD44-A2B5-) AND NOT(CD44+) AND CD133+ AND CD15+ AND NOT(A2B5+) AND CD44+ A2B5+
CD133+ only =	NOT(CD15+) AND CD44-A2B5- AND NOT(A2B5+) AND NOT(CD44+ A2B5+) AND NOT(CD44+) AND CD133+
CD133+ CD15+ =	CD133+ AND CD15+ AND NOT(CD44+) AND CD44-A2B5- AND NOT(A2B5+) AND NOT(CD44+ A2B5+)
CD133+ A2B5+ =	A2B5+ AND NOT(CD44+ A2B5+) AND NOT(CD44-A2B5-) AND NOT(CD44+) AND CD133+ AND NOT(CD15+)
CD133+ CD15+ A2B5+ =	NOT(CD44+ A2B5+) AND A2B5+ AND NOT(CD44-A2135-) AND NOT(CD44+) AND CD133+ AND CD15+
CD15+ only =	NOT(CD44+ A2B5+) AND NOT(A2B5+) AND NOT(CD44+) AND CD44-A2B5- AND NOT(CD133+) AND CD15+
CD15+ A2B5+ =	NOT(CD44+ A2B5+) AND A2B5+ AND NOT(CD44-A2B5-) AND NOT(CD44+) AND CD15+ AND NOT(CD133+)
A2B5+ only =	NOT(CD44+ A2B5+) AND NOT(CD44-A2B5-) AND A2B5+ AND NOT(CD44+) AND NOT(CD133+) AND NOT(CD15+)
negative =	NOT(CD44+ A2B5+) AND NOT(A2B5+) AND NOT(CD44+) AND CD44-A2B5- AND NOT(CD133+) AND NOT(CD15+)

Supplementary Fig. 4: Gating to phenotype of stained cell populations. A, For all samples, SSC-A and FSC-A were used to remove dead cells and debris in the preparation. This P1 population was then plotted on FSC-H and FSC-W to remove any possible attached cell. **B**, A mixture of cells either unstained or stained for one of the four phenotyping antibodies (CD44, CD133, CD15 or A2B5) were used for compensation. Uncompensated cells are shown in panel B. **C**, Single-stain mixture after compensation, this population was used to designate 6 gated populations from which Boolean logic gates could be used to calculate proportions of all 16 surface marker profiles. **D**, Quantification of marker profiles for this data is shown in graph on right. The legend shows the surface marker profiles (SMP) represented in the graph, please refer to Fig. 2 for a table with all marker profiles. **E**. All 16 Boolean logic gates used to separate cells belonging to different surface marker profiles.

Supplementary figure 5

A Generation of clonal populations from bar-coded cells (G19 C6)

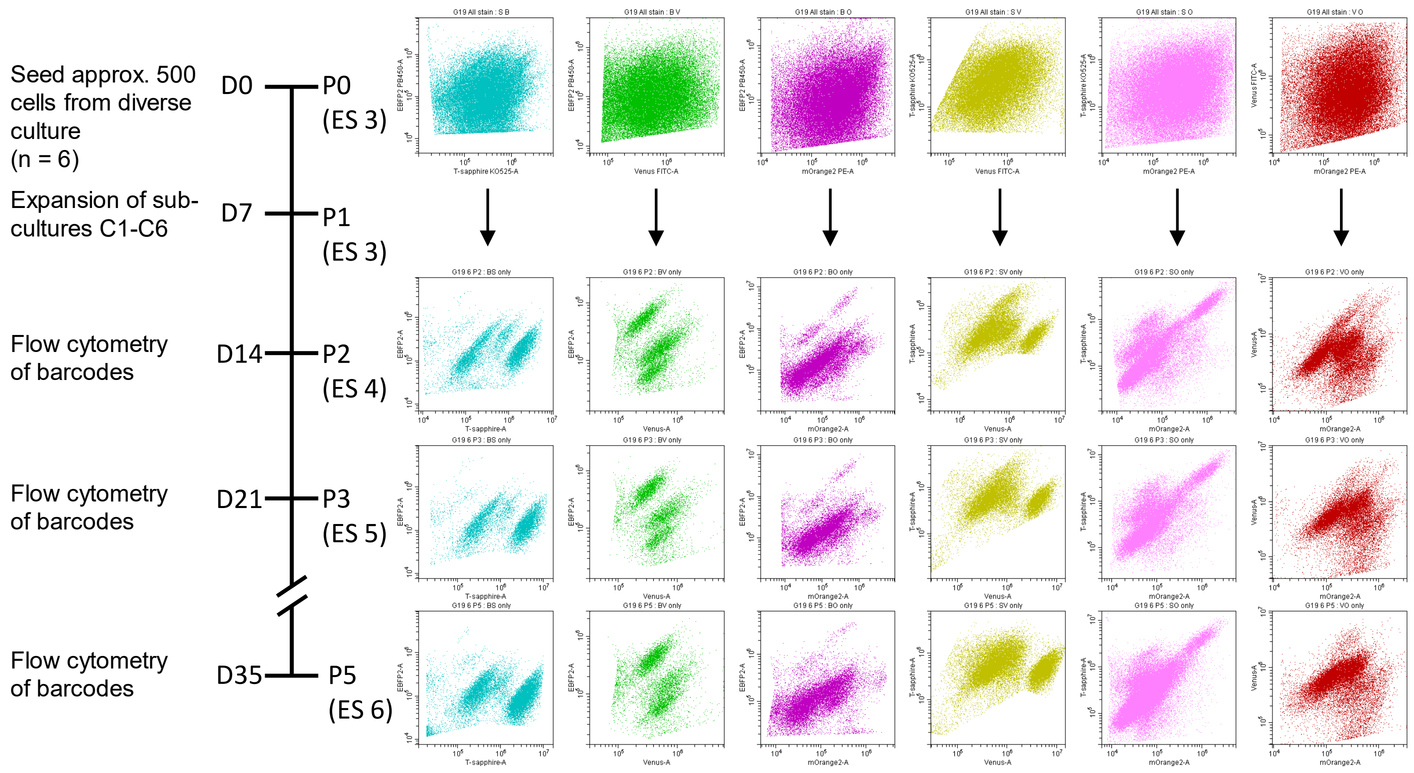

B Expression of barcode labels in the 500-cell sub-cultures of G61

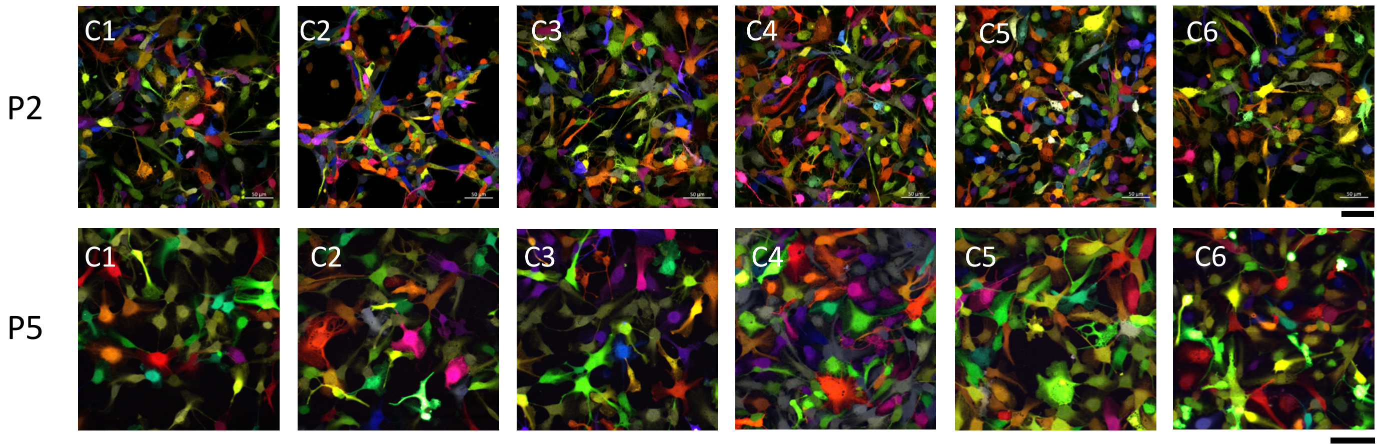

Supplementary Fig. 5: clonal populations emerge during passaging of barcode labelled cells in cell line G19. **A**, flow cytometry of barcode labelled cells before seeding at P0, (ES 2). Expansions of cells into subcultures C1-C6 and formation of clonal population visible as “clouds” or “streaks” during propagation. Clonal formation in culture C3 (ES 3-ES 6), corresponding to passages P2, P3, and P5). **B**, confocal imaging of cultures C1-C6 at P2 and P5, showing a relatively homogeneous distribution of barcoded cells (ES 4, 6). Scale bar corresponds to 50 µm

Supplementary figure 6

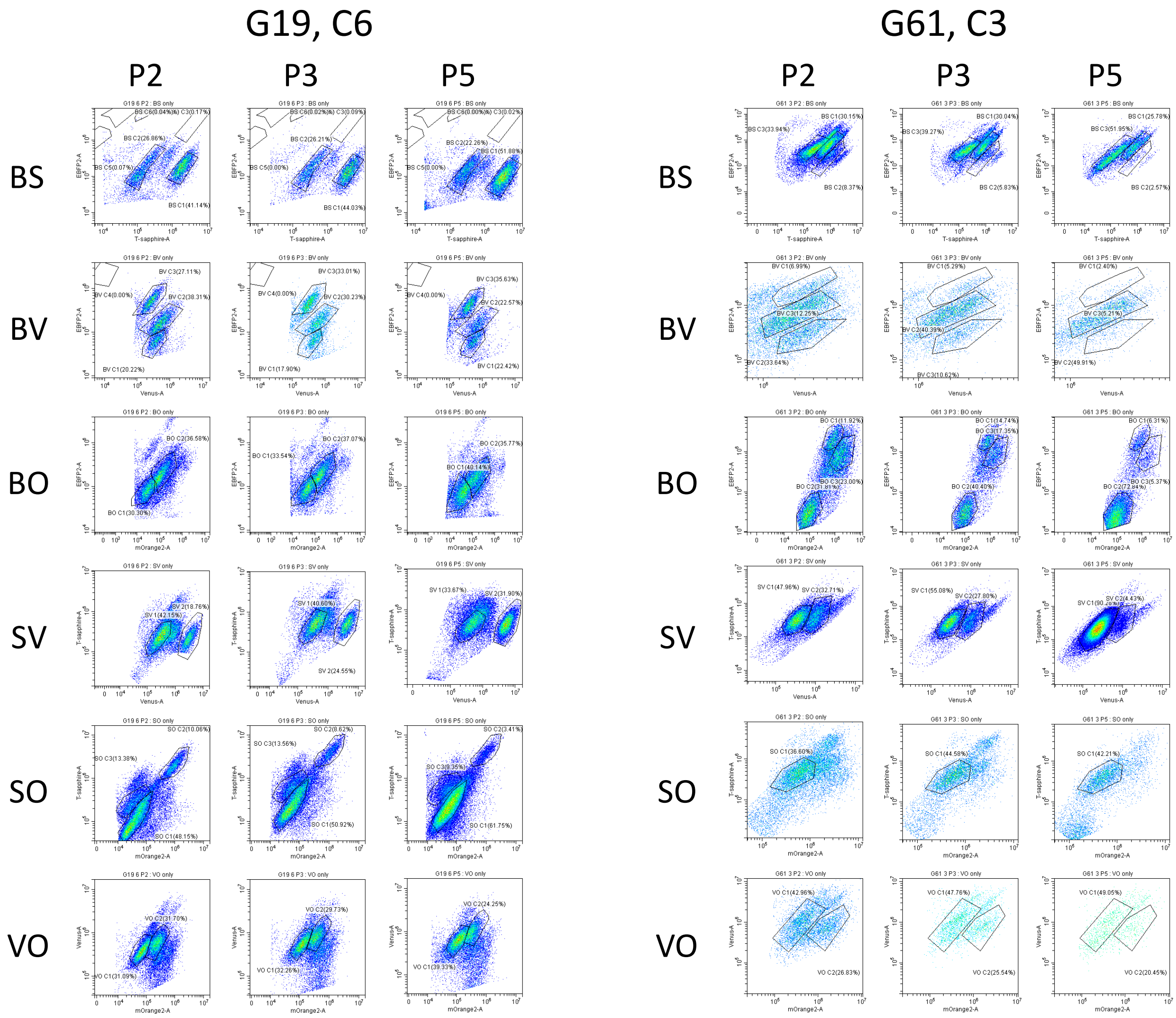

Supplementary Fig. 6: flow cytometry of barcode labelled cells to identify clonal population for selection. Each barcoded doublet (BS, BV, PO, SV, SO, VO) was identified and dominant subpopulations were "gated" to highlight clonal populations. Passaging of cells (P2, P3, and P5) show changing populations with emergence, growth and also reduction, represented by marked clusters in these flow diagrams.

Supplementary figure 7

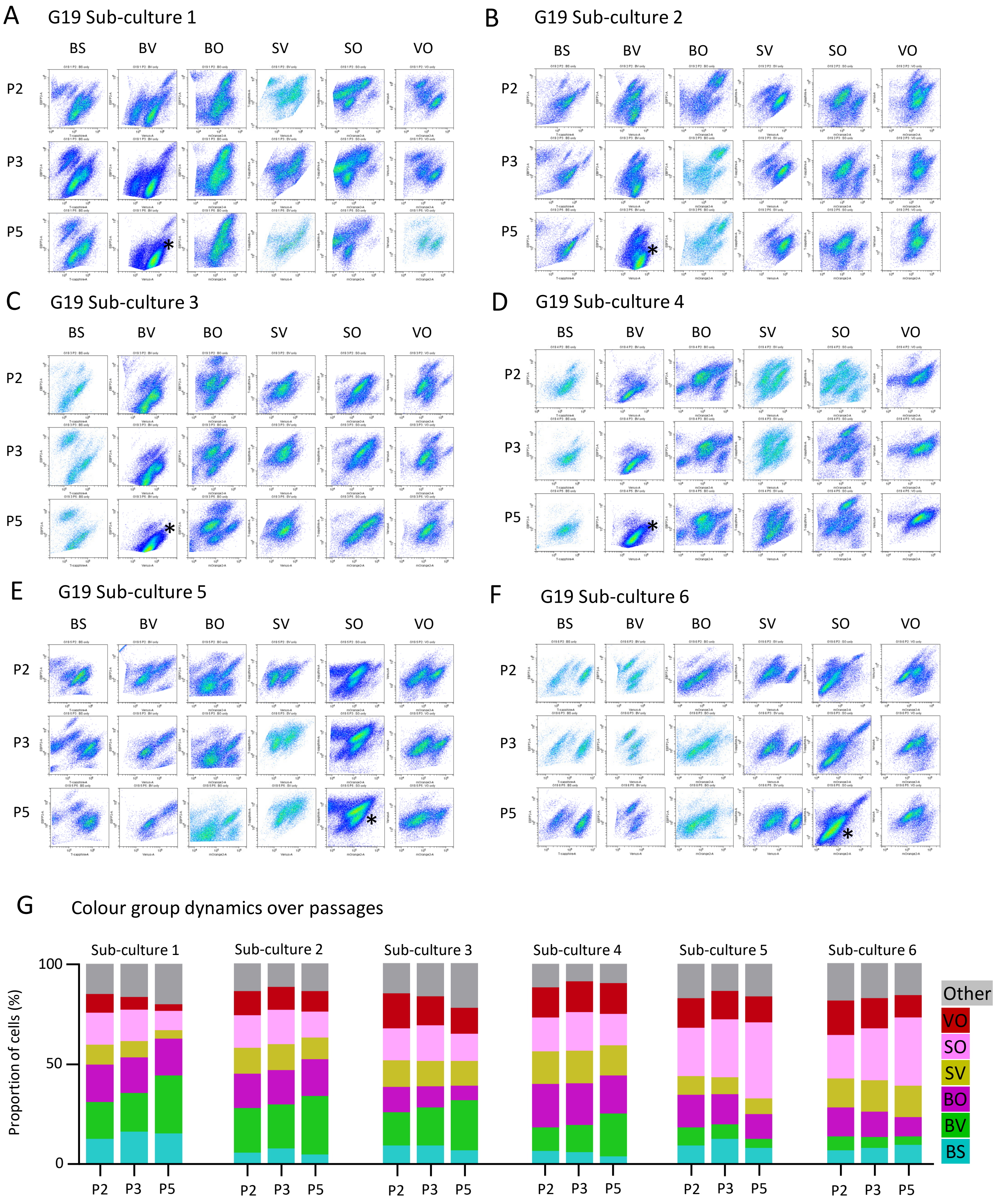

Supplementary Fig. 7: summary of all plots of the barcode doublets (BS, BV, PO, SV, SO, VO) of the 6 subcultures (A-F) of cell line G19. G, Proportional contribution of the 6 bar-coded doublet fractions to each subculture at passages P2, P3, and P5.

Supplementary figure 8

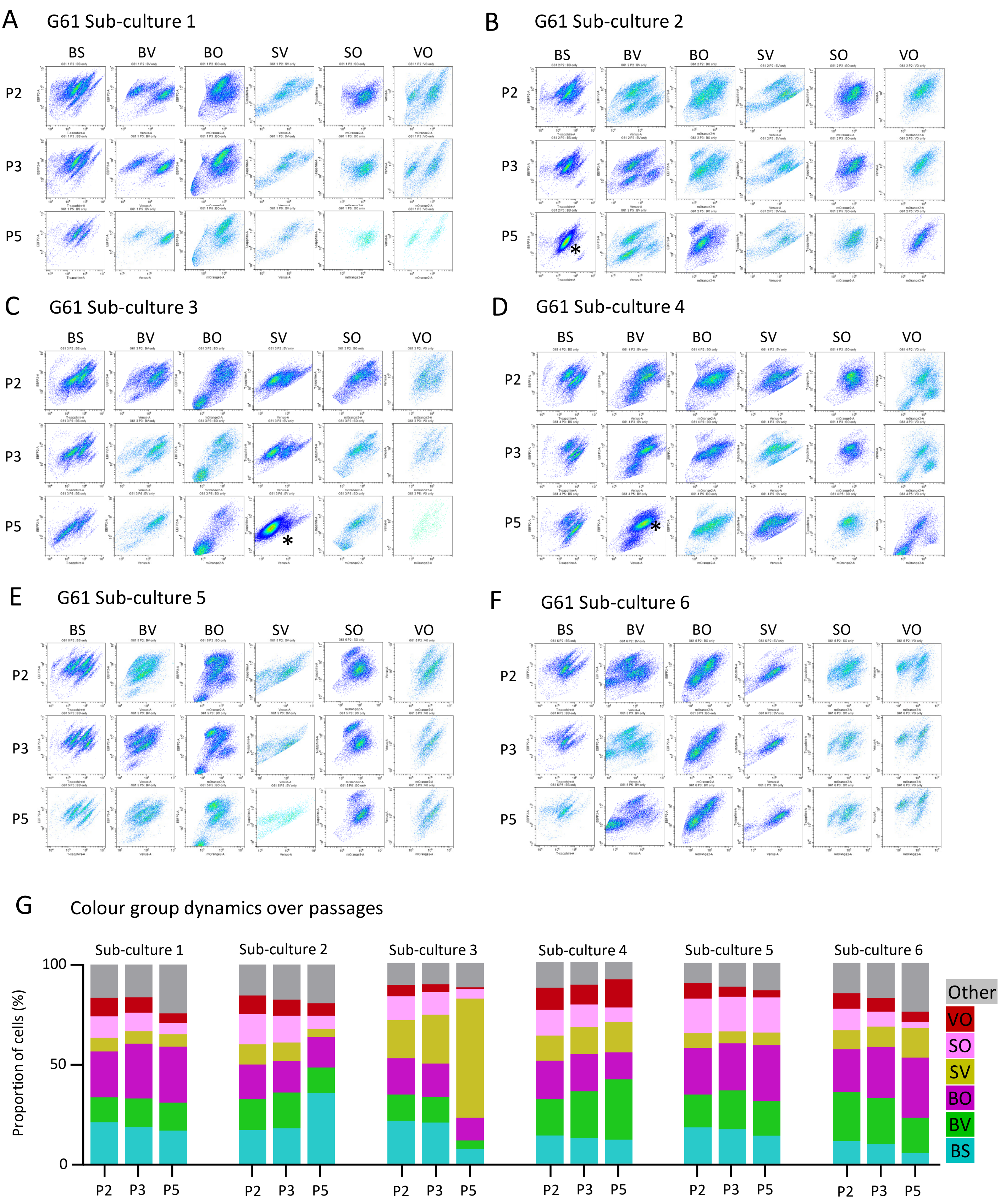

Supplementary Fig. 8: summary of all plots of the barcode doublets (BS, BV, PO, SV, SO, VO) of the 6 subcultures (A-F) of cell line G61. G, Proportional contribution of the 6 bar-coded doublet fractions to each subculture at passages P2, P3, and P5.

Supplementary figure 9

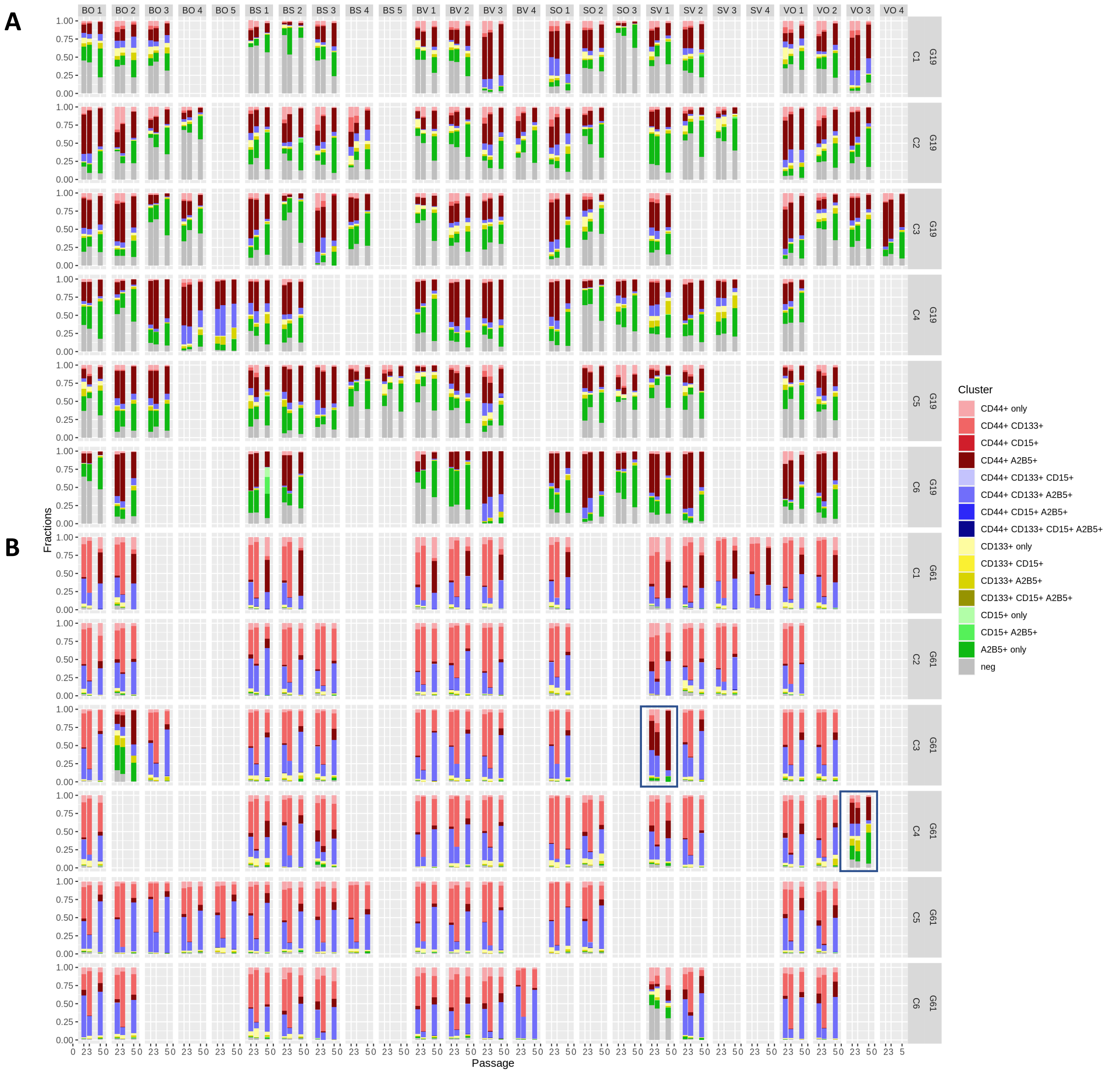

Supplementary Fig. 9: plot of the surface marker profiles (phenotypes) of barcode-label populations. Data represent all 6 subcultures C1-C6, of cell lines G19 (**A**) and G61 (**B**). The selection process of barcode doublets is shown in Supplementary Figure 2. The figure shows a selection of up to 5 barcode label doublets. Selection of fewer label doublets results in void areas. Boxed areas in G61-C3 and G61-C4 correspond to annotated populations in figure 5A.

Supplementary Figure 10

A Average for each clone’s similarity to the other clones in each culture (C1-C6)

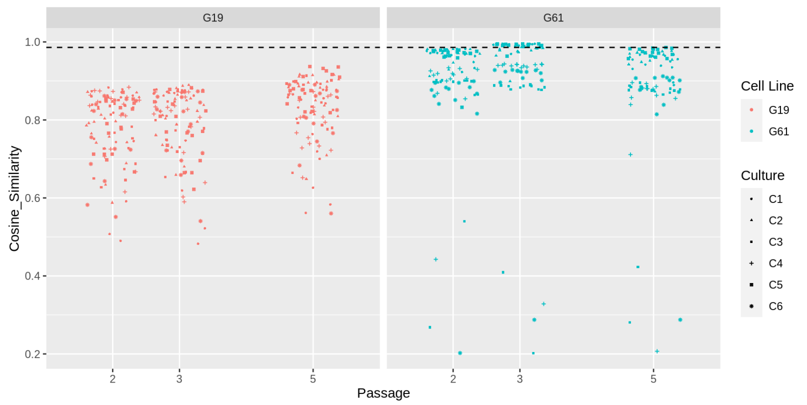

B Degree of similarity of barcode doublets across 6 cultures C1-C6, to whole cultures (range 0.5-1)

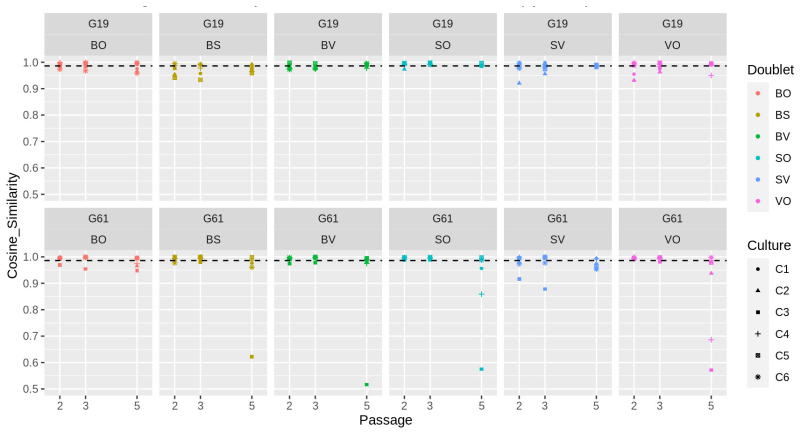

C Degree of similarity of barcode doublets across 6 cultures C1-C6, to whole cultures (range 0.9-1.0)

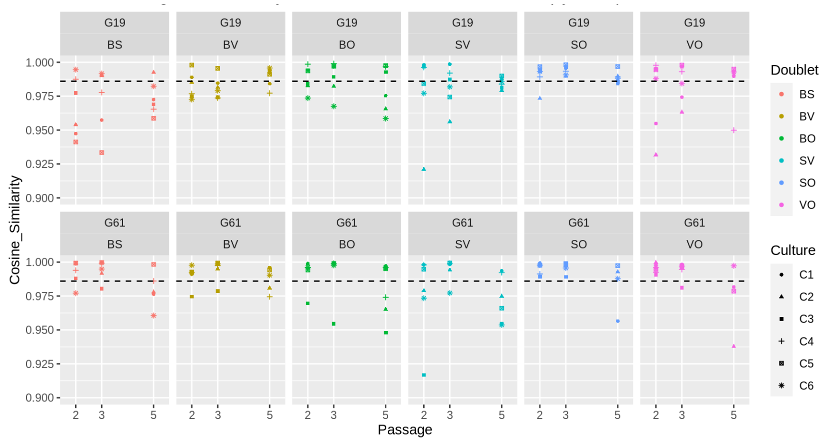

D Across-barcode doublet assessment of similarity of whole cultures within each culture and passage

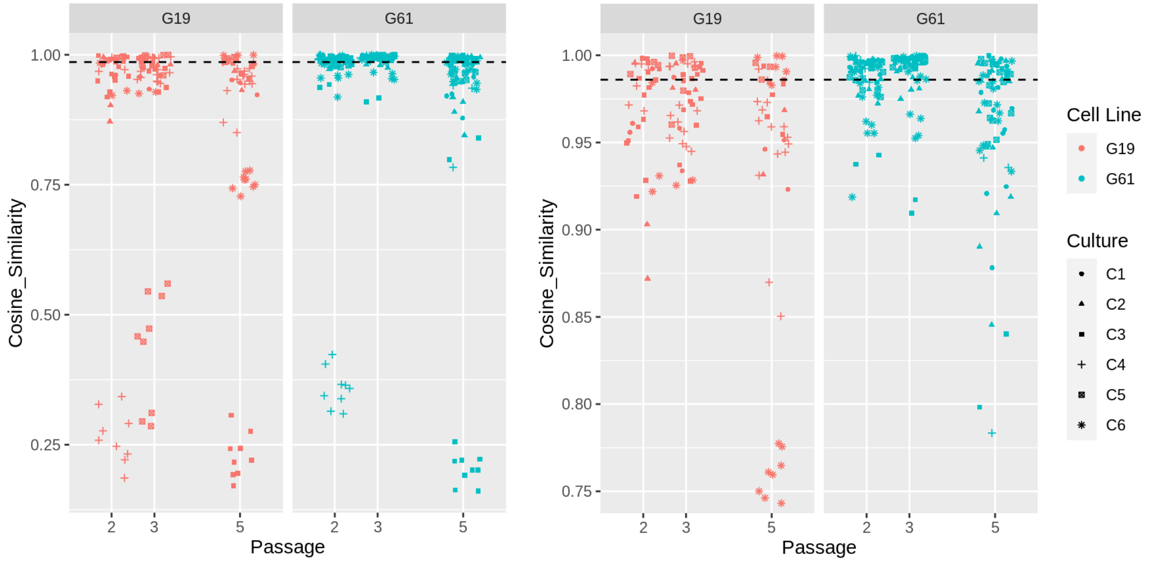

Supplementary Fig. 10: computational analysis of similarities of clones and barcode labelled doublets. A, Plot of the cosine of similarity for each clone's similarity to the other clones in each cell culture (C1-C6). Each point represents the mean of comparisons to all other clones within the same culture. G19 shows a lower degree of similarity which slightly adjusts upwards. G61 shows overall a higher degree of similarity but several clones show a very dissimilar phenotype. See also Supplementary figure 3. **B**, degree of similarity of barcode doublets across the 6 cultures C1-C6, to whole cultures. In general, the doublets appear coherent relative to the whole culture. G61 has a more doublets than G19 that are distinct from the whole culture at P5. **C**, same data with a scale ranging from 0.9-1.0 to visualise the scatter within this narrower range. **D**, left panel, assessment across barcodes of the similarity of whole cultures within each culture and passage. Broadly, the coherence of the doublet phenotypes within batches is coherent with only a few exceptions. One G19 culture each in P2 and P3 shows a loss of coherence of doublets with each other and within two batches at P5. G61 has high coherence at P2 and P3 which reduces at P5. Right panel, same data as before, "zoomed in" to the range of 0.75-1.00. **A-D**, Black dashed line – below line $p<0.001$ (based on Monte Carlo simulations).

Supplementary figure 11

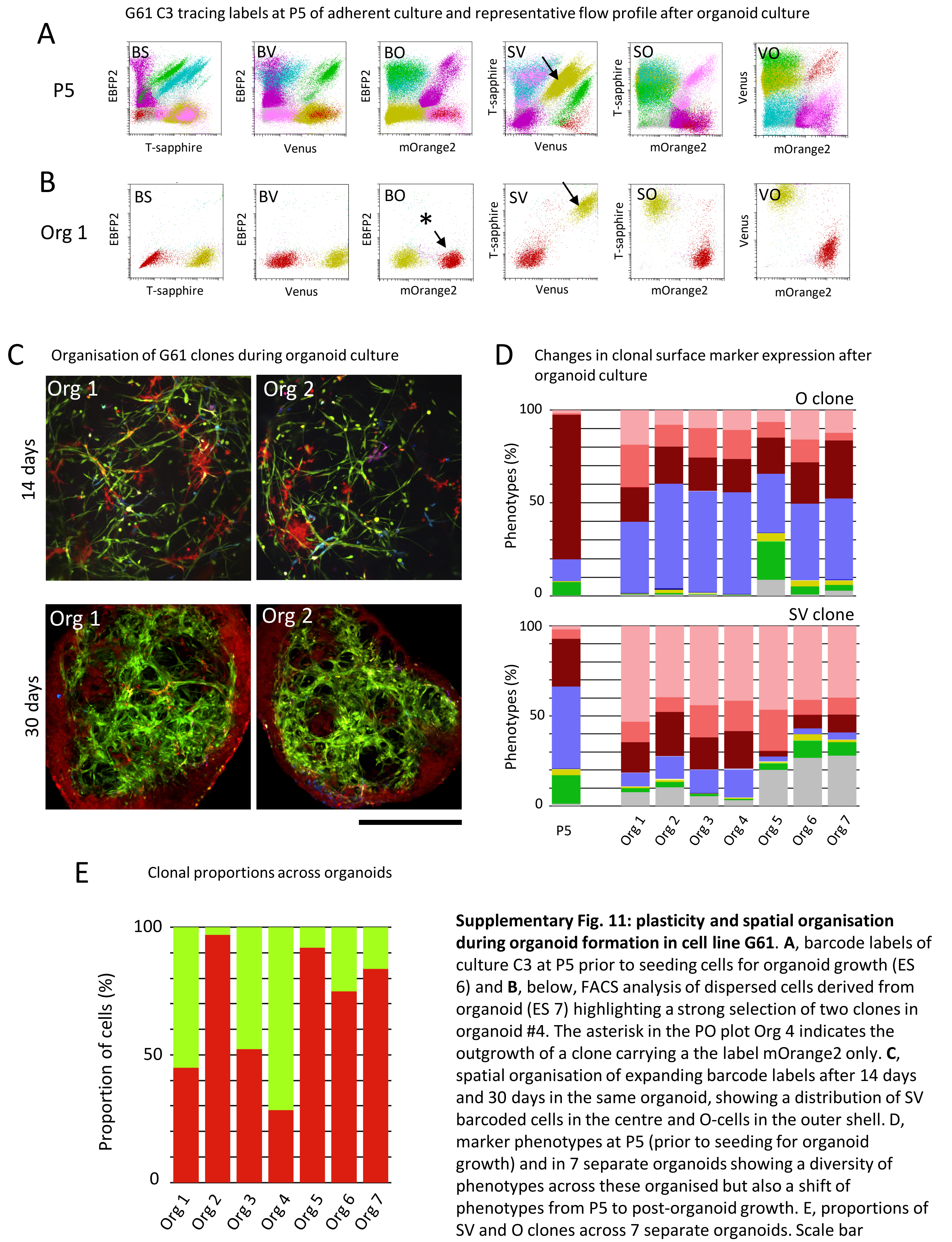

Supplementary Fig. 11: plasticity and spatial organisation during organoid formation in cell line G61. **A**, barcode labels of culture C3 at P5 prior to seeding cells for organoid growth (ES 6) and **B**, below, FACS analysis of dispersed cells derived from organoid (ES 7) highlighting a strong selection of two clones in organoid #4. The asterisk in the PO plot Org 4 indicates the outgrowth of a clone carrying a the label mOrange2 only. **C**, spatial organisation of expanding barcode labels after 14 days and 30 days in the same organoid, showing a distribution of SV barcoded cells in the centre and O-cells in the outer shell. D, marker phenotypes at P5 (prior to seeding for organoid growth) and in 7 separate organoids showing a diversity of phenotypes across these organised but also a shift of phenotypes from P5 to post-organoid growth. E, proportions of SV and O clones across 7 separate organoids. Scale bar corresponds to 200 µm in the upper images (14 days) and to 1 mm in the lower images (30 days).

Supplementary figure 12

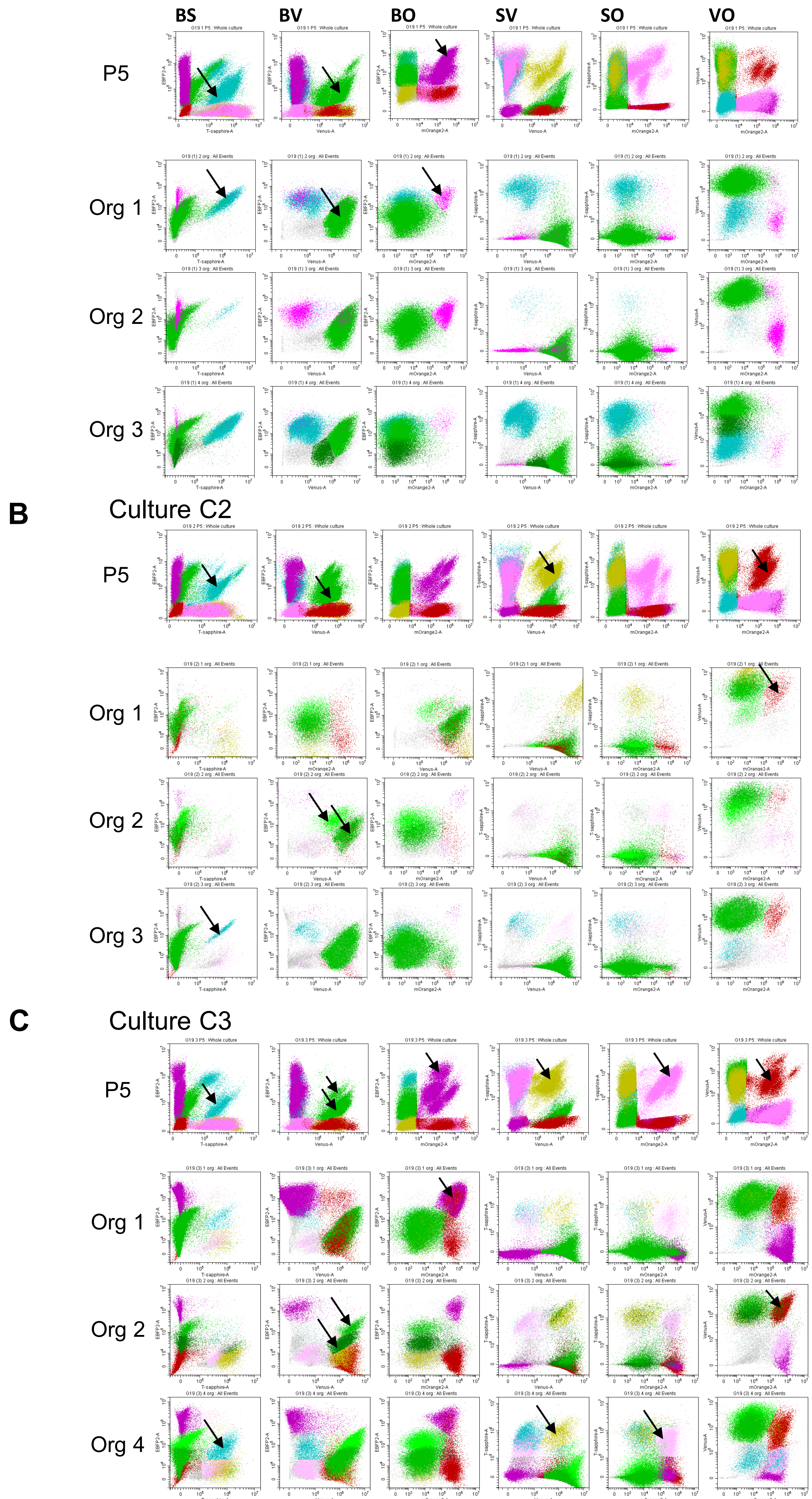

Supplementary Fig. 12: P5 clonal dominance is maintained in tumour organoids: A, flow diagrams showing barcoded population in the P5 culture of cell line G19 (C1) prior to seeding for organoid formation (top row) and analysis of barcoded cells of individual organoids (rows 2, 3, 4). Arrowheads indicate clonal populations which persist in organoids. **B**, **C,** plots of P5 culture C2 and C3, respectively, and derived organoids derived therefrom. The organoid numbers correspond to those in Supplementary Figure 9. B, organoids 1, 2, and 3 derived from subculture C to. In some of the plots there are multiple clusters in the BV barcode, and these are coloured in different shades of green. C, organoids 1, 2, and 4 derived from subculture C3.

Supplementary figure 13

A 30 day G19 organoids grown from C1, 2 and 3

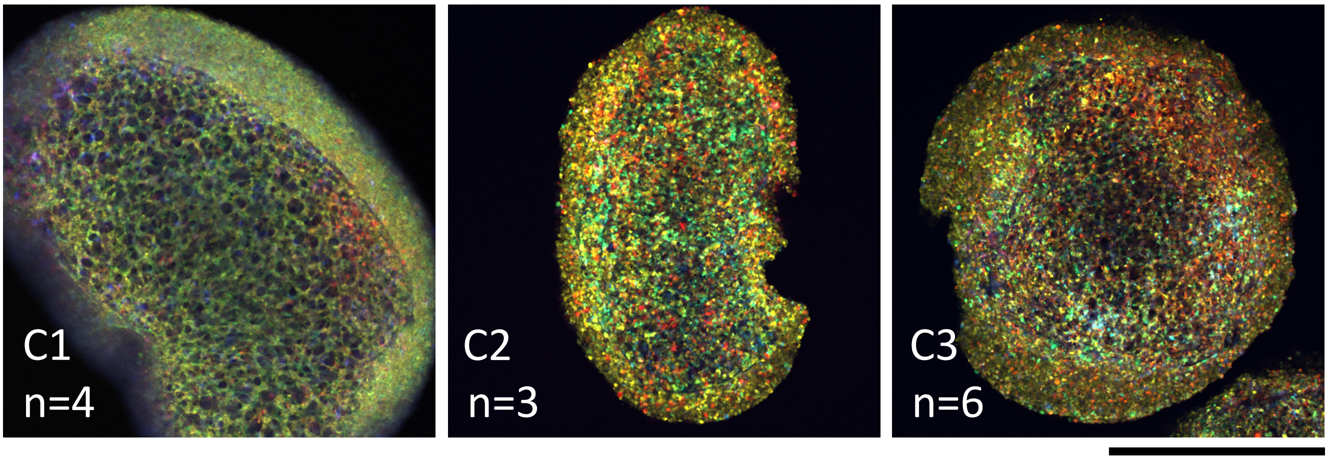

B Marker phenotypes for all clones identified in G19 organoids

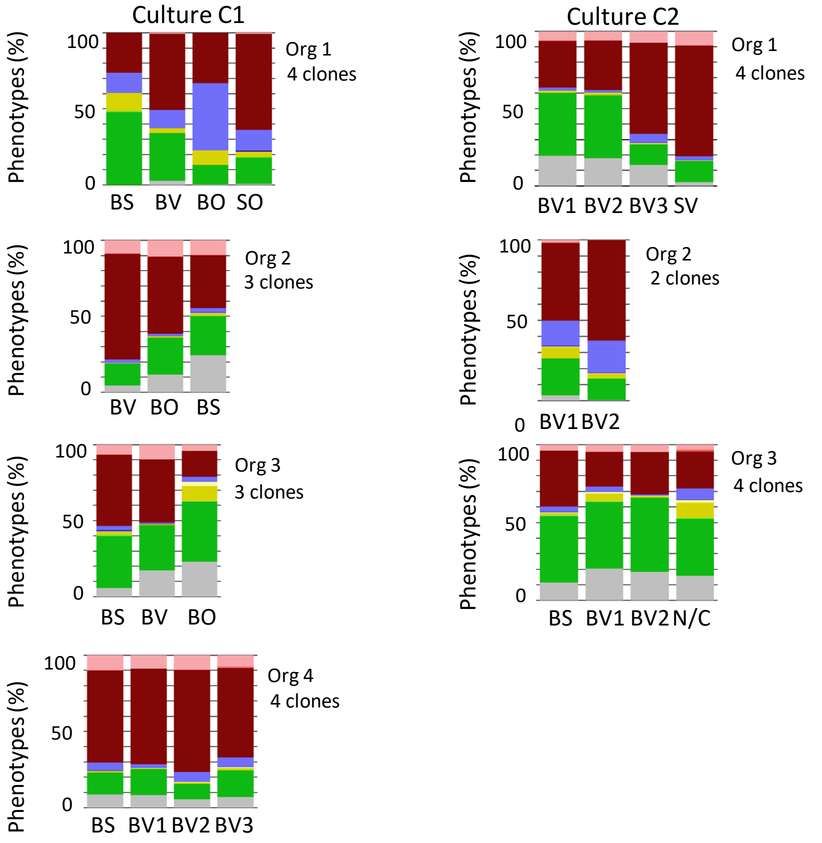

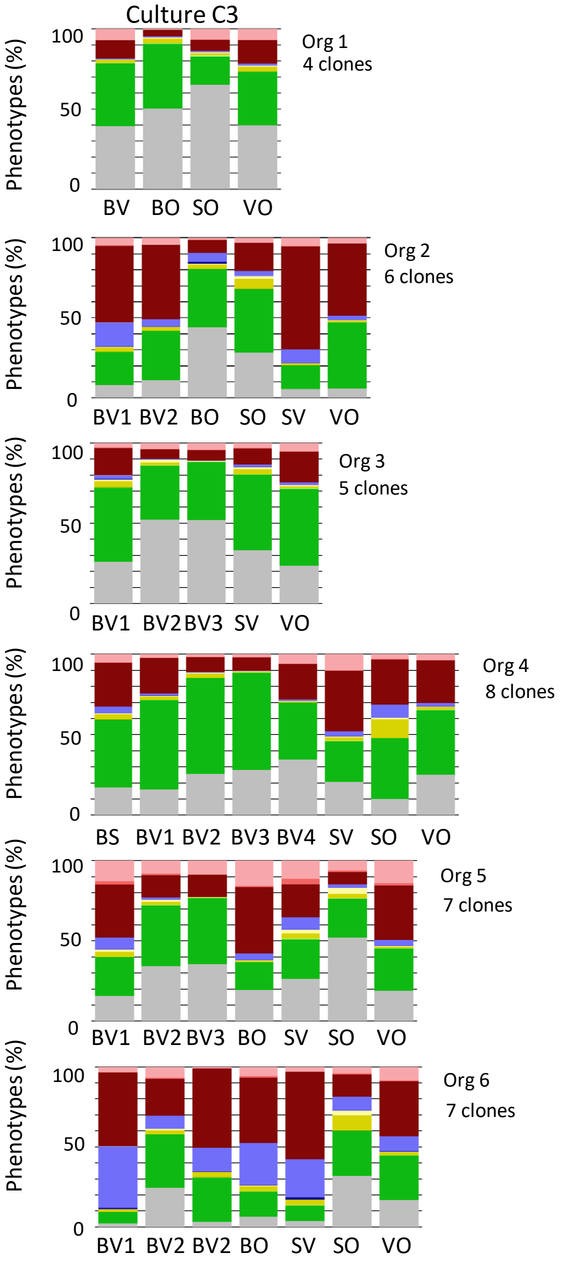

Supplementary Fig. 13: spatial distribution of barcode labelled cells and their phenotypic profile in cell line G19. A, representative examples from organoids grown from C1 (4 organoids), C2 (3 organoids), and C3 (6 organoids) at 30 days. Cells can be seen growing more diffusely than in G61. **B**, surface marker profile (phenotype) of prominently detected clones (> 400 cell detections). Unlike G61, G19 organoids grown from the same sub-culture showed variability in their clonal make-up. This is best illustrated in subculture 3 (C3) where one organoid was composed of 8 predominant clones but Org 6 composed of only 4 predominant clones. Scale bar corresponds to 1 mm.

Supplementary figure 14

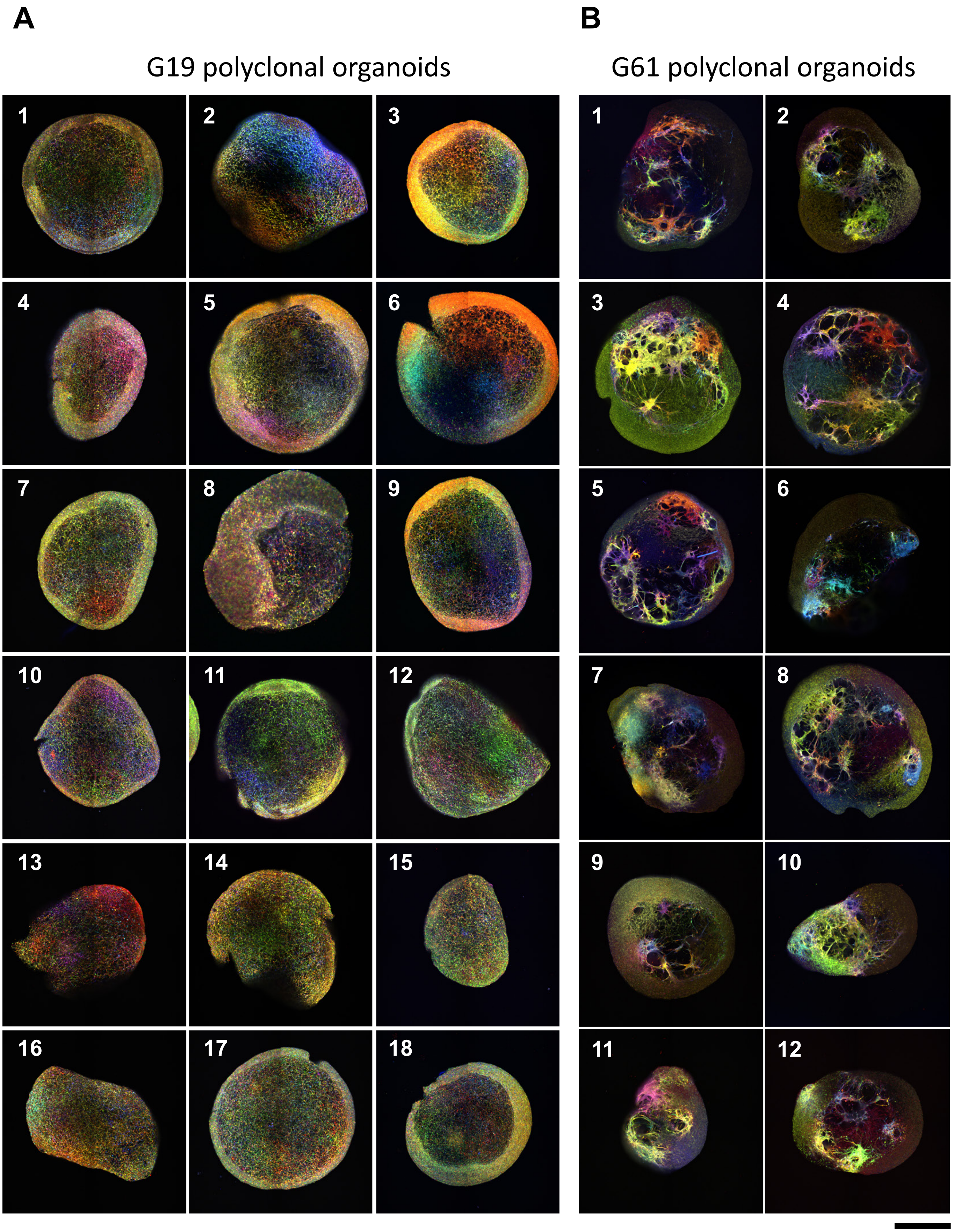

Supplementary Fig. 14: Spatial distribution of barcode labelled cells in Organoids grown from P0 labelled population. A, Organoids grown from diversely labelled P0 G19 population. B, Organoids grown from diversely labelled P0 G61 population. Each panel represents a different organoid showing a single 300µm vibratome section. Both cell lines showing a greater mix of different labels than observed in organoids grown from sub-cultured cells, but both retain a similar architecture to organoids grown from sub-cultured cells. In keeping with our previous experiments; G61 and G19 organoids show quite distinct architecture and clonal spatial distributions. G61 coloured clones show a more rigid spatial separation in discrete regions internally and externally. G19 cells however, show cluster of cells which are distributed more diffusely as a result of organoids mixing to a larger degree with regions occupied by other clones. Scale bar corresponds to 750 µm

Cell line	G19	G61
**ID**	NH15-2101	NH16-2806
**Patient age (Y)**	65	66
**Patient sex**	Female	Female
**Tumour location**	Fontal-parietal lobe	Frontal lobe
**Histology**	Glioblastoma, WHO Grade 4	Glioblastoma, WHO Grade 4
**IDH status**	Wildtype	Wildtype
**TERT status**	C228T	C228T
**MGMT promoter methylation status**	Unmethylated	Methylated
**Methylation class**	GBM IDHwt; RTK I	GBM IDHwt; RTK II
**Calibrated score**	0.97	0.94
**Copy number profile**	MYCN amp, PDGFR amp, Chr 7 gain	CDKN2A/B del, Chr 7 gain, Chr 10 loss

Supplementary table 1:

Demographic data of the patients and molecular profiles of the primary tumours from which the cell lines G19 and G61 were derived. Further context in the material and methods sections.

Protein	Fluorochrome	Abbreviation	Clone	Excitation/Emission max (nm)	Laser	Bandpass
EBFP2	EBFP2	B	-	383/448	405	450/45
T-sapphire	T-sapphire	S	-	399/511	405	525/40
Venus	Venus	V	-	515/528	488	525/40
mOrange2	mOrange2	O	-	549/565	561	585/42
CD133	BV711	-	AC133	408/711	405	710/40
CD44	APC-Vio770	-	DV105	752/775	633	780/60
A2B5	APC	-	105HB29	651/660	633	660/10
CD15	BV780	-	VIMC6	408/785	405	780/60

Supplementary table 2:

Imaging parameters: Details of excitation wavelengths of fluorescent proteins and antibody labels.

CD44	CD133	CD15	A2B5	
				SMP1
				SMP2
				SMP3
				SMP4
				SMP5
				SMP6
				SMP7
				SMP8
				SMP9
				SMP10
				SMP11
				SMP12
				SMP13
				SMP15
				SMP15
				SMP16

Supplementary table 3:

Combination of surface markers and nomenclature of surface marker profiles (SMP) SMP1-SMP16; see also shown in Figure 3.
